# The gut microbiome regulates astrocyte reaction to Aβ amyloidosis through microglial dependent and independent mechanisms

**DOI:** 10.1186/s13024-023-00635-2

**Published:** 2023-07-06

**Authors:** Sidhanth Chandra, Antonio Di Meco, Hemraj B. Dodiya, Jelena Popovic, Leah K. Cuddy, Ian Q. Weigle, Xiaoqiong Zhang, Katherine Sadleir, Sangram S. Sisodia, Robert Vassar

**Affiliations:** 1 Ken and Ruth Davee Department of Neurology, Northwestern University Feinberg School of Medicine, Chicago, IL 60611 USA; 2 Medical Scientist Training Program, Northwestern University Feinberg School of Medicine, Chicago, IL 60611 USA; 3 Department of Neurobiology, University of Chicago, Chicago, IL 60637 USA; 4 Mesulam Center for Cognitive Neurology and Alzheimer’s Disease, Northwestern University Feinberg School of Medicine, Northwestern University, Tarry Building Room 8-711, 300 East Superior Street, Chicago, IL 60611 USA

**Keywords:** Astrocyte, Neuroinflammation, Gut microbiome, Amyloid

## Abstract

**Background:**

Previous studies show that antibiotic-mediated (abx) alteration of the gut microbiome (GMB) results in a reduction of amyloid beta (Aβ) plaques and proinflammatory microglial phenotype in male APPPS1-21 mice. However, the effect of GMB perturbation on astrocyte phenotypes and microglial-astrocyte communication in the context of amyloidosis has not been examined.

**Methods:**

To study whether the GMB modulates astrocyte phenotype in the context of amyloidosis, APPPS1-21 male and female mice were treated with broad-spectrum abx leading to GMB perturbation. GFAP + astrocytes, plaque-associated astrocytes (PAA), PAA morphological parameters, and astrocyte complement component C3 levels were quantified using a combination of immunohistochemistry, immunoblotting, widefield microscopy, and confocal microscopy. Furthermore, these same astrocyte phenotypes were assessed in abx-treated APPPS1-21 male mice that received either fecal matter transplant (FMT) from untreated APPPS1-21 male donors to restore their microbiome or vehicle control. To assess complete absence of the GMB on astrocyte phenotypes, the same astrocyte phenotypes were quantified in APPPS1-21 male mice raised in germ-free (GF) or specific-pathogen free conditions (SPF). Lastly, we assessed whether microglia are necessary for abx-induced astrocyte phenotypes by depleting microglia in APPPS1-21 male mice via treatment with a colony-stimulating factor 1 receptor (CSF1R) inhibitor (PLX5622) and vehicle control or PLX5622 and abx.

**Results:**

Herein, we demonstrate that postnatal treatment of male APPPS1-21 mice with broad-spectrum abx leading to GMB perturbation reduces GFAP + reactive astrocytes and PAAs, suggesting that the GMB plays a role in regulating reactive astrocyte induction and recruitment to Aβ plaques. Additionally, we show that compared to controls, PAAs in abx-treated male APPPS1-21 mice exhibit an altered morphology with increased number and length of processes and reduced astrocytic complement C3, consistent with a homeostatic phenotype. GFAP + astrocyte reduction, PAA reduction, astrocyte morphological changes, and C3 levels are restored when abx-treated mice are subject to FMT from untreated APPPS1-21 male donor mice. Next, we found that APPPS1-21 male mice raised in GF conditions have similar astrocyte phenotypes as abx-treated male APPPS1-21 male mice. Correlational analysis revealed that pathogenic bacteria depleted by abx correlate with GFAP + astrocytosis, PAAs, and astrocyte morphological changes. Finally, we determined that abx-mediated reduction in GFAP + astrocytosis, PAAs, and astrocytic C3 expression is independent of microglia. However, abx-induced astrocyte morphological alterations are dependent on the presence of microglia, suggesting that there is both microglial independent and dependent GMB control of reactive astrocyte phenotypes.

**Conclusions:**

We show for the first time, in the context of amyloidosis, that the GMB plays an important role in controlling reactive astrocyte induction, morphology, and astrocyte recruitment to Aβ plaques. GMB regulation of these astrocytic phenotypes is both independent and dependent on microglia.

**Supplementary Information:**

The online version contains supplementary material available at 10.1186/s13024-023-00635-2.

## Background

Alzheimer’s disease (AD) is a progressive neurodegenerative disorder that is the most common cause of dementia in the elderly for which there are few effective disease-modifying therapies [1]. AD is characterized pathologically by the presence of amyloid beta (Aβ) plaques and neurofibrillary tau tangles in the brain. Additionally, neuroinflammation in the form of microgliosis and astrogliosis is a crucial hallmark of AD [1–3]. Recent genome-wide association studies have identified numerous AD genetic risk factors that are associated with innate immunity [4–9]. However, the mechanisms governing glial activation in the context of AD pathogenesis are still unclear. Greater understanding of these mechanisms and the role of glia in AD could lead to the development of novel disease modifying therapies.

Astrocytes are an abundant glial cell type present in the brain that play an important role in brain homeostasis. They function in neurotransmitter recycling, ionic buffering, regulation of synaptic transmission, and maintenance of the blood brain barrier [10]. Astrocytes are also key players in the neuroinflammation of many neurodegenerative diseases [11–15]. Homeostatic astrocytes are converted into a neurotoxic reactive astrocyte subtype by microglial release of interleukin 1ɑ, TNFɑ, and complement factor C1q [11]. Reactive astrocytes, defined by unique morphology and gene expression profile, lose normal homeostatic functions, can kill neurons, and potentially modulate Aβ deposition in AD models [16]. Furthermore, recent astrocyte-enriched single-cell RNA sequencing datasets suggest that there are most likely several astrocyte subtypes that modulate the progression of neurodegenerative diseases and are context-specific [17–19]. A recent study showed that astrocytic gene expression and morphological changes in response to pathology are related. This study also found that astrocytes in AD APP/PS1 mice have reduced morphological complexity and territory size compared to wildtype mice, which is associated with upregulation of astrocytic expression of proinflammatory and AD-risk genes and a downregulation of homeostatic genes. Furthermore, CRISPR-Cas9 deletion of the genes *Ezrin* and *Fermt2*, which strongly correlated with astrocyte morphology and territory size in wildtype mice, resulted in reduced astrocyte territory size, performance on novel object recognition task, and colocalization of synaptic markers PSD95 and VGLUT1. This suggests that astrocyte morphology is likely important in regulating astrocytic function, cognition, and brain homeostasis [20].

The gut microbiome (GMB), comprised of trillions of bacteria that inhabit the human intestine, has been implicated in numerous neurological disease etiologies, such as Multiple Sclerosis, Parkinson’s disease, and AD [21–31]. In AD, it was previously shown that patients that are amyloid positive have an increased amount of pro-inflammatory cytokines in their blood and a higher abundance of *Escherichia*/*Shigella* in the gut compared to healthy controls [32]. Another study has shown a decrease in gut *Firmicutes* and *Bifidobacterium* and an increase in *Bacteroidetes* in human AD patients compared to healthy controls [33]. These studies indicate there is likely a difference in the GMB between AD patients and healthy controls, which may drive pathological progression of AD. In mouse models of amyloidosis, there is a shift in GMB composition compared to wildtype mice [34–40]. Furthermore, perturbation of the GMB via a short-term abx cocktail causes a reduction in Aβ plaque deposition in the brains of APPPS1-21 male mice, a well-studied animal model of AD [26, 27]. Additionally, abx treatment causes a shift in microglial reactivity from disease associated microglial (DAM) morphological and transcriptomic phenotype to a homeostatic phenotype [26, 27]. Aβ deposition and DAM phenotype were restored to control levels when abx-treated mice received FMT to restore their GMB from mice with an unperturbed GMB. These results suggest that abx-induced reductions of Aβ pathology is mediated by the GMB and that the GMB can influence microglia, which can in turn influence Aβ deposition.

Microglial depletion via CSF1R inhibition demonstrated a failure of abx mediated GMB perturbation to lower amyloidosis in APPPS1-21 mice [26, 27]. This suggests that microglia are important for the abx-mediated reduction in amyloidosis. Furthermore, it has been shown that APPPS1-21 and 5XFAD mice raised in germ-free conditions, which do not develop a GMB, have a reduction in amyloidosis and alteration of microglial phenotypes [34, 41]. These studies indicate that the complete absence of a GMB has a similar effect on amyloidosis and microglial phenotypes compared to abx-mediated GMB perturbation. In addition to amyloidosis, the GMB has recently been shown to be important in the progression of tau pathology and tau-mediated neurodegeneration in an APOE and sex-dependent manner. Abx treatment and germ-free conditions reduced tau pathology, neuroinflammation, and neurodegeneration [42]. Although the effect of GMB perturbation on microglial response in AD amyloidosis has been explored, the role of the GMB in modulating astrocyte phenotypes has not been investigated.

Herein, we have investigated the effect of GMB manipulation on astrocyte phenotypes in APPPS1-21 mice. We report that postnatal abx treatment of APPPS1-21 male mice results in a decrease in GFAP + astrocytes and PAAs at 9 weeks and 3 months of age. Additionally, we observed changes in PAA morphology and a decrease in astrocyte complement component C3. Specifically, in terms of morphology, we found that abx treatment in APPPS1-21 male mice reduces the size of astrocytic cell bodies while increasing the number and length of the cellular processes when compared to vehicle treated APPPS1-21 male mice. However, there is no difference in astrocyte morphological phenotypes in abx-treated female APPPS1-21 mice compared to vehicle-treated female mice, similar to previous studies that assessed microglial phenotypes in this paradigm [26, 27]. Additionally, we found that FMT restores abx-mediated changes in GFAP + astrocytosis, PAAs, astrocyte morphology, and astrocyte C3 levels, suggesting that the observed astrocyte phenotypic changes are indeed a result of GMB manipulation. We then verified abx-mediated changes in astrocyte phenotypes in male APPPS1-21 mice that were raised in germ-free (GF) conditions. GF APPPS1-21 mice have changes in astrocyte phenotypes similar to abx mice when compared with mice raised in a specific pathogen free (SPF) environment. We performed correlational analysis and found that bacteria depleted by abx treatment positively correlate with GFAP + astrocytosis and PAAs and negatively correlate with astrocyte process number and length. These findings suggest that this group of bacteria may mediate astrocyte reaction to Aβ plaque pathology in our model. Lastly, we studied whether GMB-mediated regulation of astrocyte phenotypes is microglial dependent or independent by treating APPPS1-21 male mice with vehicle control and CSF1R inhibition or abx and CSF1R inhibition. We found that abx-mediated reduction in GFAP + astrocytosis, PAAs and astrocytic C3 expression is independent of microglia. However, we found that abx-induced morphological changes are dependent on microglia. Overall, our results suggest that the GMB has a role in activating astrocyte-mediated neuroinflammation in AD and show for the first time the presence of a GMB-amyloid-astrocyte axis. Additionally, we find that GMB-mediated control of reactive astrocyte takes place through both microglial independent and dependent mechanisms.

## Methods

### Animal housing and handling

APPPS1-21 mice [43] on a C57BL/6 J background were obtained from Mathias Jucker (German Center for Neurodegenerative Diseases, DZNE). Mice were housed in a specific pathogen free environment in the Center for Comparative Medicine at Northwestern University. 9-week abx treatment experiments were conducted at Northwestern, All experimental procedures were approved by the IACUC office of Northwestern University. Germ-free APPPS1-21 animals were generated in the gnotobiotic mouse facility at the University of Chicago. Specific pathogen free APPPS1-21 animals were housed in conventional environments in the University of Chicago Animal Resources Center. These mice are demonstrated to be free of disease-causing pathogens that could affect mouse health. FMT, GF, and PLX experiments were conducted at the University of Chicago. All experimental procedures for these mice were approved by the IACUC office of University of Chicago.

### Antibiotics treatment

APPPS1-21 male and female mice were orally gavaged with 200 µL of an antibiotic cocktail (4 mg/ml kanamycin, 0.35 mg/ml gentamicin, 8,500 U/ml colistin, 2.15 mg/ml metronidazole, 0.45 mg/ml vancomycin in autoclaved water) or water vehicle from postnatal day (PND) 14 to PND 21 as previously described [27]. During abx treatment, cages were changed everyday to avoid accumulation and consumption of old feces. Mice were transcardially perfused at 9 weeks or 3 months of age with perfusion buffer (20 mg/ml phenylmethylsulfonyl fluoride, 5 mg/ml leupeptin, 200 nM sodium orthovanadate, and 1 M dithiothreitol in 1X PBS). Brains were harvested and weighed. Left hemibrains were fixed in 10% formalin and cryopreserved in 30% sucrose/1X PBS for immunofluorescence microscopy analysis and right hemibrains were dissected into cortex, hippocampus and cerebellum (control) and flash frozen in LN2 for biochemical analysis. Hemibrains were coronally sectioned on a Epredia HM 430 freezing-sliding microtome into 40 µm sections.

#### PLX5622 treatment

Male APPPS1-21 male mice were treated with abx or water vehicle from PND 14–21 as described above. These animals then all received a PLX5622 infused diet at a dose of 1200 ppm from PND 24 until sacrifice at 3 months. PLX5622 was obtained from Plexxikon and added in the AIN-76 diet by Research Diets Inc.

### Fecal matter transplant experiment

Fecal slurries were prepared daily by collecting fresh fecal matter pellets (5 mg) from age-matched untreated APPPS1-21 male mice and mixing with 1 mL of sterile water. The feces were homogenized in the water using a dounce homogenizer. After allowing the suspension to settle for 5 min the supernatant was collected. After slurry preparation, 200 µL of fecal slurry or water vehicle were administered to abx-treated (PND 14–21) APPPS1-21 male mice via oral gavage from PND24–9 weeks.

### Immunohistochemistry

4 comparable 40 µm coronal mouse brain sections containing cortex were chosen to perform IHC. Sections were washed 3 times in TBS buffer for 5 min with mild shaking and then incubated in 16 mM glycine / 0.25% triton TBS solution for 1 h at room temperature. After 3 × 5-min TBS washes, sections were incubated in 5% donkey serum / 0.25% triton TBS solution for 1 h. After 3 × 5-min triton TBS washes, sections were incubated in donkey anti-mouse IgG (Additional file 10). After additional 3 × 5-min triton TBS washes, sections were incubated overnight at 4°C with primary antibodies diluted in 1% BSA / 0.25% triton TBS solution (1% BSA buffer) (Additional file 10). The following day, sections were rinsed 3 × for 10 min in 1% BSA buffer. Fluorescently labeled secondary antibodies in 1% BSA buffer were added to the sections for 1 h at room temperature in the dark (Additional file 10). After 3 × 5-min TBS washes, sections were mounted on diamond white glass microscope slides (cat # 1358W, Globe Scientific Inc.) using Prolong Gold antifade reagent (cat #P36930, Invitrogen) and 24 × 40 mm No 1.5 gold seal cover glasses (cat # 3421, Thermo Scientific).

### Microscopy

Widefield and confocal imaging was performed in Northwestern University Center for Advanced Microscopy. Widefield images were taken on a Nikon Ti2 widefield microscope with a 10 × air objective (0.3 numerical aperture) using NIS Elements software. Images were saved as ND2 files. After widefield images were taken, using Nikon Elements software, cortex was outlined using the simple ROI polygon tool. ND2 images were then analyzed using Nikon Elements General Analysis tool for GFAP + astrocytes, Aβ plaques, and plaque-associated astrocytes (where at least 1 pixel of GFAP signal contacts 1 pixel of Aβ signal). This tool was also used for Sox9 and S100B signal analysis. Mean Aβ plaque size was calculated by dividing Aβ plaque area signal by number of Aβ plaques detected.

Confocal z-stack images were taken using a Nikon A1R (Figs. 2 and 4) and AXR (Figs. 6, 8 and 9) confocal microscope at 40 × oil objective (1.3 numerical aperture) with a 1.5 × zoom for 25–30 µm total stacks with each step of the z-plane being 0.5 µm using NIS Elements software. Three microenvironments in the cortex were taken per animal. Each microenvironment contained an astrocyte localized to an amyloid plaque unless there was no instance where this occurred in the cortex of the section. Confocal and widefield images were acquired and analyzed in a blinded manner.

### Astrocyte IMARIS analysis

For astrocyte 3D reconstruction, confocal z-stacks were imported into IMARIS. Maximum projection confocal images were used to define C3, GFAP, and Aβ plaque signal for each microenvironment. Using the filaments tool, the IMARIS software calculated mean process length, area, volume, soma volume, number of processes, number of process branch points, and number of process terminal points. C3 area and GFAP area were calculated using the 3D reconstructions for each microenvironment.

### Dodiya et al. (2022) section analysis

Sections used in Figs. 3, 4, 8 and 9 were taken from studies performed in Dodiya et al. 2022 [27]. Sections were freshly stained as described in the IHC methodology section. Microbiome data and methodologies used to generate tissue from these groups can be found in Dodiya et al. 2022 [27].

### Immunoblotting

Cortices were weighed and homogenized in radioimmunoprecipitation assay buffer (10 µl/mg) (RIPA) (50 mM tris, 0.15 M NaCl, 1% octylphenoxypolyethoxyethanol (IGEPAL), 0.1% SDS, and 0.5% sodium deoxylate at pH 8) containing protease (cat # 535140, Calbiochem) and phosphatase inhibitor (cat # 78427 Thermo Fisher Scientific) cocktails. Samples were kept on ice for 30 min following homogenization and then sonicated on ice for 20 s each. Following sonication, the samples were centrifuged at 4 °C/14,000 RPM and supernatant was then taken for downstream analysis. Protein concentration was determined using the Pierce BCA Protein Assay kit (cat # 23225, Thermo Scientific). Lysates were diluted to 2 µg/µl and for western blots 18 µg of total protein was mixed with 4 × Laemmli buffer (cat # 1610747, Bio-Rad) and heated at 95 °C for 10 min. The samples were then run on 4–12% Criterion XT Bis–Tris polyacrylamide gels (cat # 3450126, Bio-rad) in MES buffer. Gels were transferred to 0.45 µm nitrocellulose membranes (cat # 1620167, Bio-Rad) using the Bio-rad Trans-blot Turbo Transfer System. Membranes were briefly incubated in 0.1% ponceau solution to assess transfer quality. After 3 × 5 min washes in TBST, membranes were incubated in 5% milk in TBST for 1 h. Primary antibodies were incubated in 5% milk overnight at 4 °C, and secondary antibodies were incubated in 5% milk for 1 h at room temperature (antibodies listed in Additional file 10). After two TBST and one TBS 5 min washes, membranes were incubated in SuperSignal West Pico Plus Chemilumeniscent substrate (cat # 34580, Thermo Scientific). Membranes were imaged on a Bio-rad imager and analyzed using ImageLab software.

### Microbiome analysis

DNA extractions were performed on fecal pellets from male and female abx and vehicle control treated APPPS1-21 mice by the University of Illinois at Chicago Genome Research Core (UIC GRC). The V4 region of 16 s rRNA gene was amplified using the 515f and 806r primers. Sequencing was performed using paired-end 150 base reads on an Illumina MiniSeq sequencing Platform. Subsequent data analysis was performed by the University of Illinois at Chicago Research Informatics Core (UIC RIC).

### Basic processing

Forward and reverse reads were be merged using PEAR v0.9.6 [44]. Merged reads were trimmed with cutadapt v1.18 to remove ambiguous nucleotides, primer sequences, and trimmed based on quality threshold of *p* = 0.01 [45]. Reads that lacked either primer sequence or were less than 225 bp were discarded. Chimeric sequences were identified and removed using the USEARCH v8.1.1861 algorithm with a comparison to Silva v132 reference sequence [46, 47]. Amplicon sequence variants (ASVs) were identified using DADA2 v1.18 [48]. The representative sequences for each ASVs were then annotated, taxonomically using the Naïve Bayesian classifier included in DADA2 with the Silva v132 training set.

### Differential analysis of microbial taxa

Differential analyses of taxa as compared with experimental covariates (*i.e.,* treatment status and gender) were performed using the software package edgeR on raw sequence counts [49]. Prior to analysis the data were filtered to remove any sequences that were annotated as chloroplast or mitochondria in origin as well as removing taxa that had less than 1,000 total sequence counts or were present in less than 20% of the samples. Data were normalized as counts per million. Normalized data were then fit using a negative binomial generalized linear model using the experimental covariates and statistical tests were performed using a quasi-likelihood ratio test, i.e. glmQLFTest function in edgeR. Additional pairwise tests of the experimental groups were performed using the exactTest function in edgeR. In all cases, adjusted *p* values (q values) were calculated using the Benjamini–Hochberg false discovery rate (FDR) correction [50]. The complete differential analysis is available in Additional file 11.

### Alpha diversity analyses

Shannon indices were calculated with default parameters in R using the vegan library [51]. Prior to analysis, the data were rarefied to a depth of 5,000 counts per sample. The resulting Shannon indices were then modelled with the experimental covariates using a generalized linear model (GLM) assuming a Gaussian distribution. Significance of the model (ANOVA) was tested using the F test. Post-hoc, pairwise tests were performed using Mann–Whitney test. Plots were generated in R using the ggplot2 library.

### Beta diversity/Dissimilarity analyses

Bray–Curtis indices were calculated with default parameters in R using the vegan library. Prior to analysis the normalized data were square root transformed. The resulting dissimilarity indices were modelled and tested for significance with the experimental covariates using the ANOSIM test. Plots were generated in R using the ggplot2 library.

### Transcriptional analysis

Genes upregulated in astrocytes in the context of amyloidosis (Jiwaji et al., 2022) [52], LPS (Zamanian et al. 2012) [53], MCAO (Zamanian et al. 2012) [53], pan-reactivity (Zamanian et al. 2012) [53], aging (Clarke et al. 2018) [54] and downregulated in aging (Clarke et al. 2018) [54] were investigated in the Dodiya et al. 2022 [27] abx vs vhl male bulk RNAseq dataset [27]. For the amyloidosis astrocyte gene list, we only considered genes increased by greater than onefold to specifically assess those genes which are highly amyloid induced in astrocytes. LPS, MCAO, and pan-reactive astrocyte gene lists were taken from Jiwaji et al. [52], which reordered the initial datasets from Zamanian et al. 2012 [53]. Briefly, to generate the LPS and MCAO datasets, Jiwaji et al. [52] required the gene to be ranked in the top 100 genes for one stimulation paradigm and at least 50 places lower in the other paradigm. To generate the pan-reactive gene list, Jiwaji et al. required genes to be in top 250 genes and no more than 50 ranking positions difference between LPS and MCAO datasets. The Dodiya et al. 2022 [27] dataset was generated from an experiment in which the authors performed bulk RNAseq from 9 weeks old APPPS1-21 male abx and vhl treated mouse cortices (*N* = 6/group). Volcano plots using these data were generated by plotting the negative Log of false discovery rate adjusted *p*-value against the log2 fold change. Representative gene changes were verified with qPCR.

### Quantitative polymerase chain reaction (qPCR)

RNA was taken from Dodiya et al. 2022 RNAseq experiment [27] to verify RNAseq hits. RNA was converted to cDNA using Invitrogen Superscript IV VILO mastermix kit (cat #11766050). Mastermixes for qPCR were made by using TaqMan fast advanced master mix (cat #4444556) and TaqMan single tube gene expression assay primers against *H2-d1* (ThermoFisher Mm04208018_gH), *Emp1* (ThermoFisher Mm00515678_m1), and *Cxcl10* (ThermoFisher Mm00445235_m1). 90 ng of template cDNA was used for each reaction. Experimental primers were normalized to *Gapdh* (ThermoFisher Hs02786624). Reactions were run on a 96 well plate in an Applied Biosystems QuantStudio 7 flex machine (cat #448701) in the Northwestern NUSeq core facility. The protocol used for amplification contained a 2-min hold step at 50 °C, a 2-min hold step at 95 °C, and 40 cycles of 1 s at 95 °C followed by 20 s at 60 °C.

### Correlational analysis between normalized bacterial counts and astrocyte phenotypes

Normalized bacterial genera counts per million from vehicle and abx treated male APPPS1-21 mice were correlated with corresponding astrocyte phenotype quantitative data in Fig. 7 (GFAP + astrocytes/µm^2^, PAAs/µm^2^, PAA %area, astrocyte process number, and astrocyte process length). The bacteria used in this analysis were those that were significantly altered by abx treatment in APPPS1-21 male mice (*Odoribacter, Muribaculum, Paraprevotella, Prevotellaceae, Millionella, Lachnospiraceae GCA-900066575, Tyzzerella,* and *Intestinimonas, Anaeroplasma, and Akkermansia).* Pearson’s correlation coefficients and two-tailed *p*-values were calculated using GraphPad Prism 9 and are available in Additional file 12.

### Statistics

**Fig. 1:**
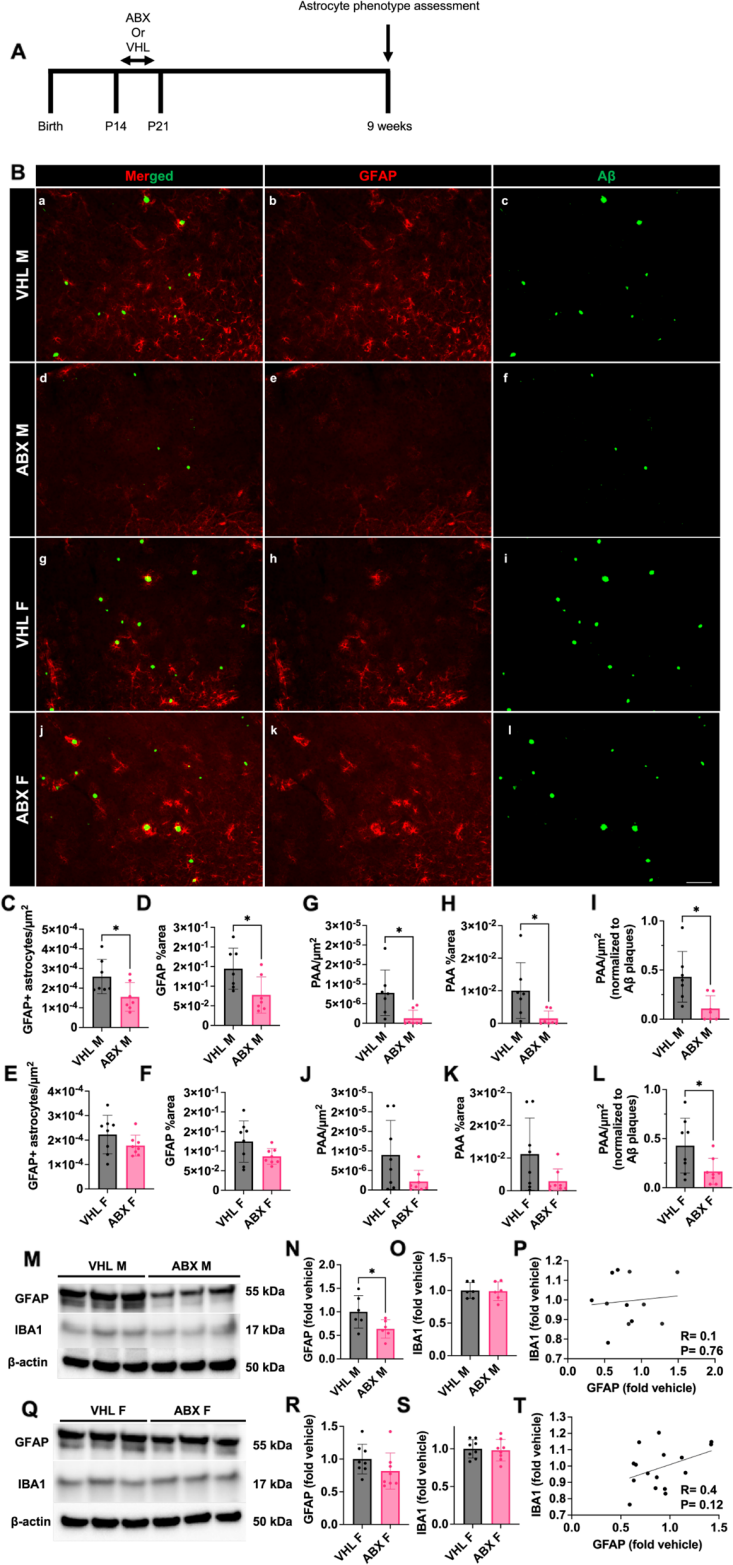
Administration of short-term antibiotics reduces GFAP + astrocytes and plaque-associated astrocytes in the brain of male APPPS1-21 mice. (A) Experimental schematic. (B) Representative merged (a, d, g, j), GFAP (b, e, h, k) and Aβ (c, f, i, l) immunosignals from vehicle (VHL) treated male (a-c), antibiotic (ABX) treated male (d-f), VHL female (g-i), and ABX female (j-l) mice. (C) Quantification of GFAP + astrocytes/µm^2^. (D) GFAP + astrocyte percent area. (E) Quantification of GFAP + astrocytes/µm^2^. (F) GFAP + astrocyte percent area. (G) Plaque-associated astrocyte /µm^2^. (H) Plaque-associated astrocytes percent area. (I) Plaque-associated astrocyte/µm^2^ normalized to Aβ plaques/µm^2^ in VHL male and ABX male APPPS1-21 mice. (J) Plaque-associated astrocytes/µm^2^. (K) Plaque-associated astrocytes percent area. (L) Plaque-associated astrocytes/µm^2^ normalized to Aβ plaques/µm^2^ in VHL female and ABX female APPPS1-21 mice. Representative male VHL and ABX GFAP, IBA1, and β-actin immunoblots (M), quantifications (N, O), and GFAP vs IBA1 correlation (P). Representative female VHL and ABX GFAP, IBA1, and β-actin immunoblots (Q), quantifications (R, S), and GFAP vs IBA1 correlation (T). Immunoblot quantifications are from full blots depicted in Supplemental Fig. 5. M = male, F = female. Data expressed as mean ± standard deviation. *N* = 6–8/group Statistics calculated using two-tailed unpaired student’s t-tests. 4 sections used per animal. * denotes a *p*-value ≤ 0.05, ** indicates *p*-value ≤ 0.01, *** indicates *p*-value ≤ 0.001, and **** indicates a *p*-value of ≤ 0.0001. Scale bar indicates 100 µm

**Fig. 2:**
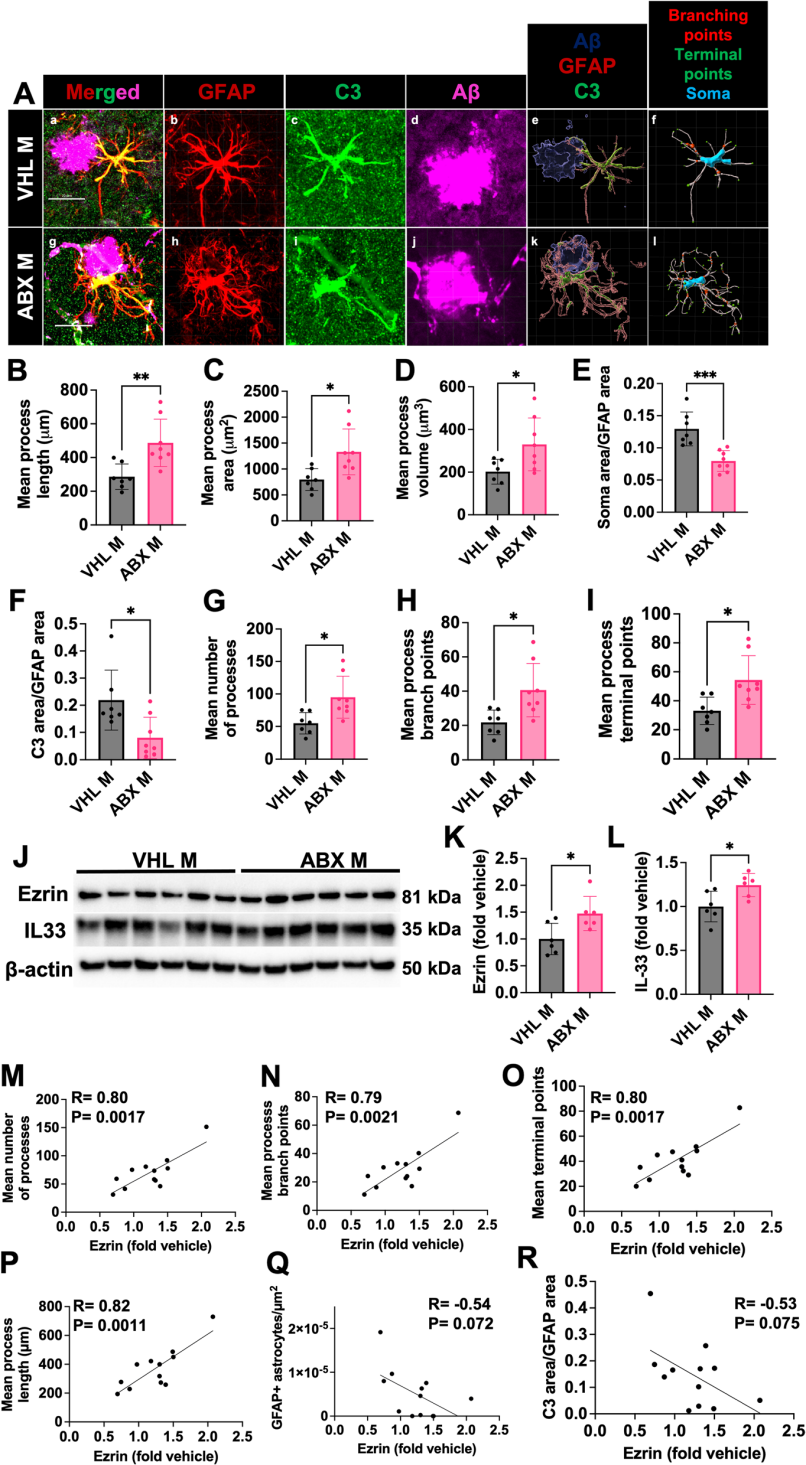
Administration of short-term antibiotics alters astrocyte morphology and C3 + astrocyte reactivity in the brain of male APPPS1-21 mice. (A) Representative GFAP, C3, and Aβ merged astrocyte z-stack maximum projections (a, g), IMARIS 3D reconstructions (e, k), and IMARIS filament 3D reconstructions (f, l) for VHL male (a-f) and ABX male (g-l) groups. GFAP (b, h), C3 (c, i), and Aβ (d, j) shown as separate channels from merged images. (B) Quantification and comparison of astrocyte mean process length sum (µm). (C) Astrocyte mean process area sum (µm^2^). (D) Astrocyte mean process volume sum (µm^3^). (E) Astrocyte soma area/GFAP area. (F) Astrocyte C3 area/GFAP area. (G) Astrocyte mean number of processes. (H) Astrocyte mean number of process branch points. (I) Astrocyte mean number of process terminal points between VHL male and ABX male groups. (J) Immunoblots for ezrin, IL33, and β-actin in ABX and VHL treated male mice. Quantifications of for ezrin (K) and IL33 (L) immunoblots normalized to β-actin in ABX and VHL treated male mice. Correlations between Ezrin protein levels and mean number of astrocyte processes (M), branch points (N), terminal points (O), process length sums (P), GFAP + astrocytes (Q), and C3/GFAP levels (R). M = male. Data expressed as mean ± standard deviation. *N *= 6–7/group. Statistics calculated using two-tailed unpaired student’s t-tests. 4 sections used per animal. * denotes a *p*-value ≤ 0.05, ** indicates *p*-value ≤ 0.01, *** indicates *p*-value ≤ 0.001, and **** indicates a *p*-value of ≤ 0.0001. Scale bars indicate 20 µm

**Fig. 3:**
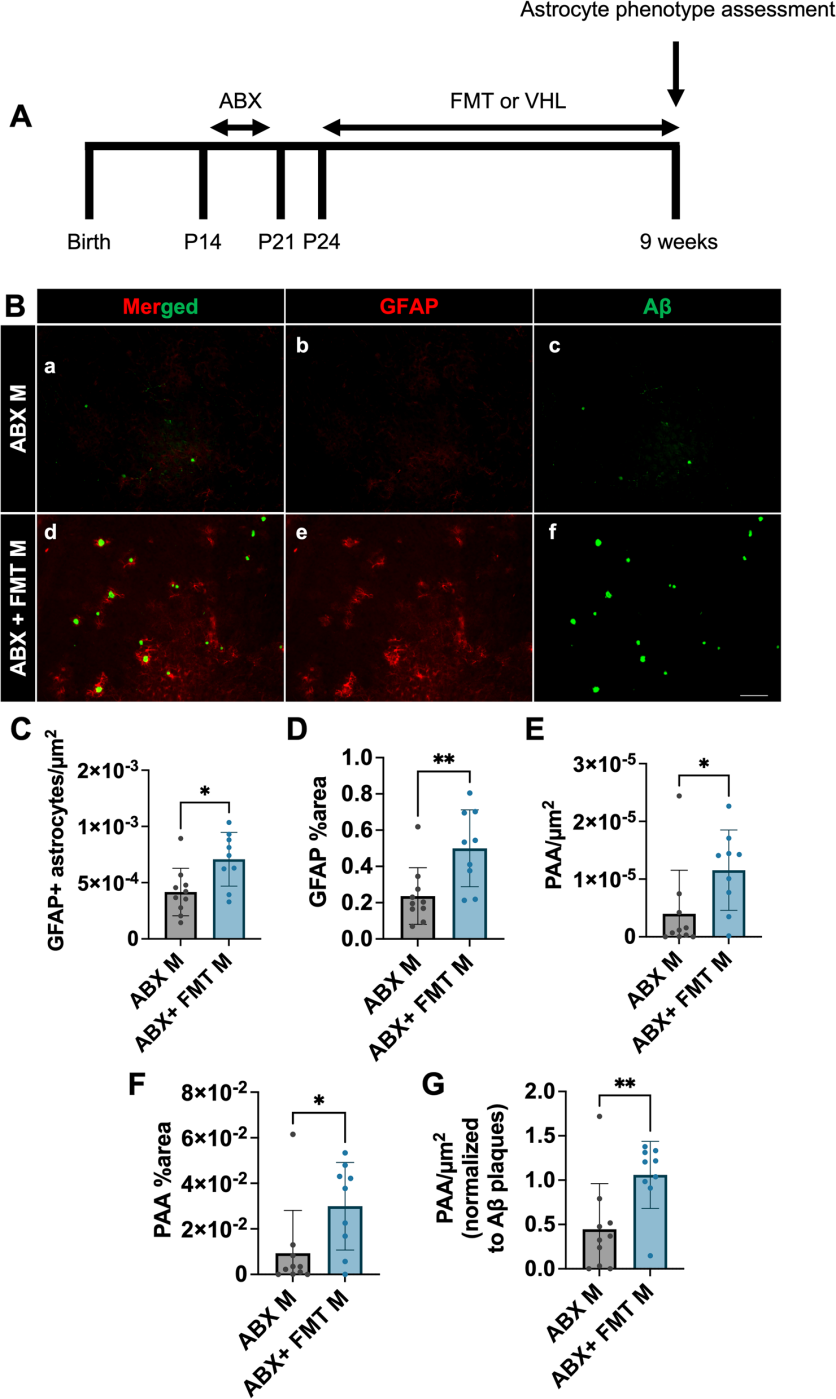
Fecal matter transplant restores GFAP + astrocytes and PAA in the brains of male APPPS1-21 mice. (A) Experimental schematic. (B) Representative merged (a, d), GFAP (b, e) and Aβ (c, f) immunosignal images from ABX M (a-c) and ABX + FMT M (d-f). (C) Quantification of GFAP + astrocytes/µm^2^. (D) GFAP + astrocyte percent area. (E) Plaque-associated astrocytes/µm^2^. (F) Plaque-associated astrocyte percent area. (G) Plaque-associated astrocytes/µm^2^ normalized to Aβ plaques/µm^2^ in ABX male and ABX-FMT male APPPS1-21 mice. M = male. Data expressed as mean ± standard deviation. ABX M N = 10, ABX + FMT M N = 9. Statistics calculated using two-tailed unpaired student’s t-tests. 4 sections used per animal. * denotes a *p*-value ≤ 0.05, ** indicates *p*-value ≤ 0.01, *** indicates *p*-value ≤ 0.001, and **** indicates a *p*-value of ≤ 0.0001. Scale bar indicates 100 µm

**Fig. 4:**
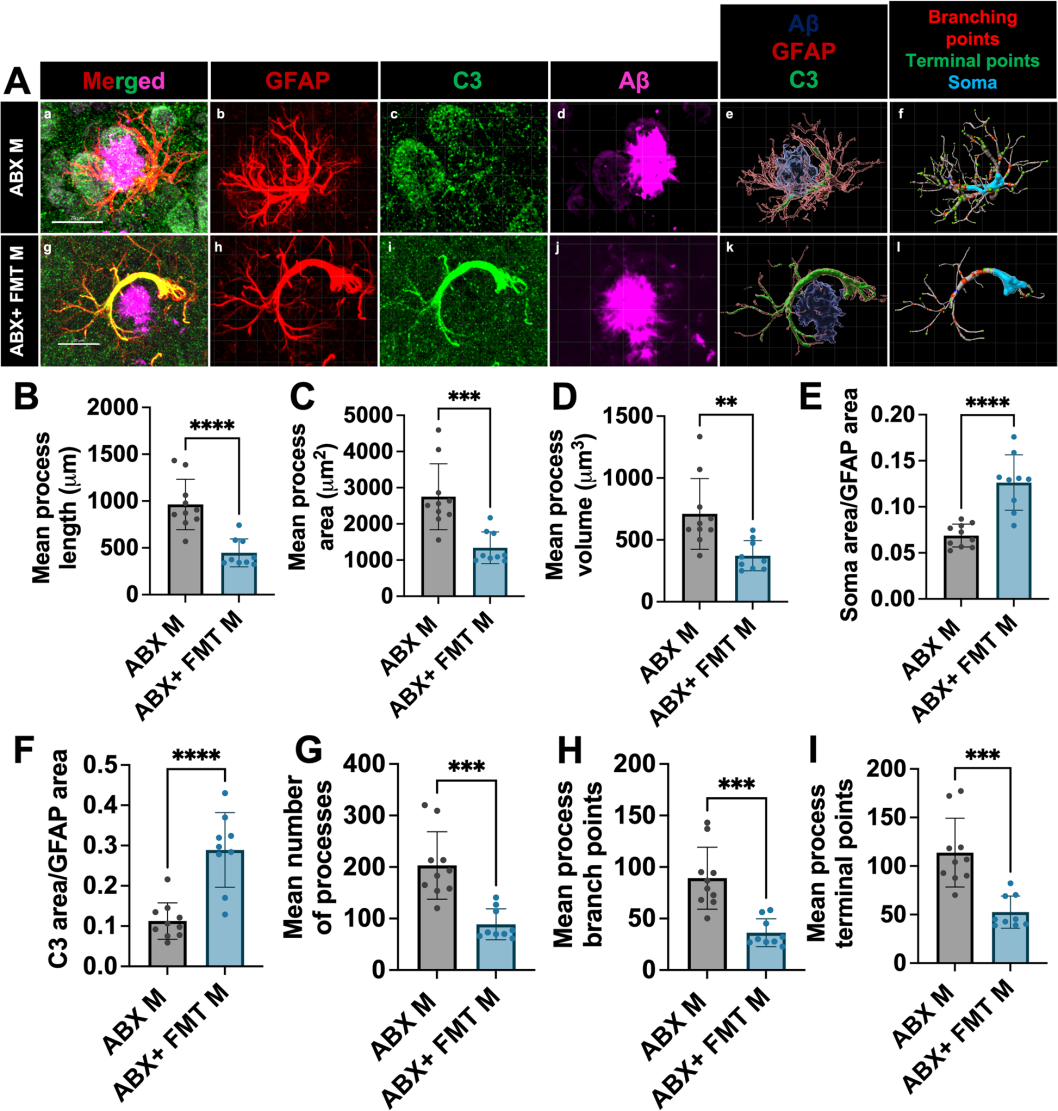
Fecal matter transplant restores astrocyte morphology and C3 + astrocyte reactivity in the brain of male APPPS1-21 mice. (A) Representative GFAP, C3, and Aβ merged astrocyte z-stack maximum projections (a, g), IMARIS 3D reconstructions (e, k), and IMARIS filament 3D reconstructions (f, l) for ABX male (a-f), and ABX + FMT male (g-k). GFAP (b, h), C3 (c, i), and Aβ (d, j) shown as separate channels from merged images. (B) Quantification and comparison of astrocyte mean process length sum (µm). (C) Astrocyte mean process area sum (µm^2^). (D) Astrocyte mean process volume sum (µm^3^). (E) Astrocyte soma area/GFAP area. (F) Astrocyte C3 area/GFAP area. (G) Astrocyte mean number of processes. (H) Astrocyte mean number of process branch points. (I) Astrocyte mean number of process terminal points between ABX male and ABX + FMT male. M = male. Data expressed as mean ± standard deviation. ABX M N = 10, ABX + FMT M N = 9. Statistics calculated using two-tailed unpaired student’s t-tests. 4 sections used per animal. * denotes a *p*-value ≤ 0.05, ** indicates *p*-value ≤ 0.01, *** indicates *p*-value ≤ 0.001, and **** indicates a *p*-value of ≤ 0.0001. Scale bars indicate 20 µm

**Fig. 5:**
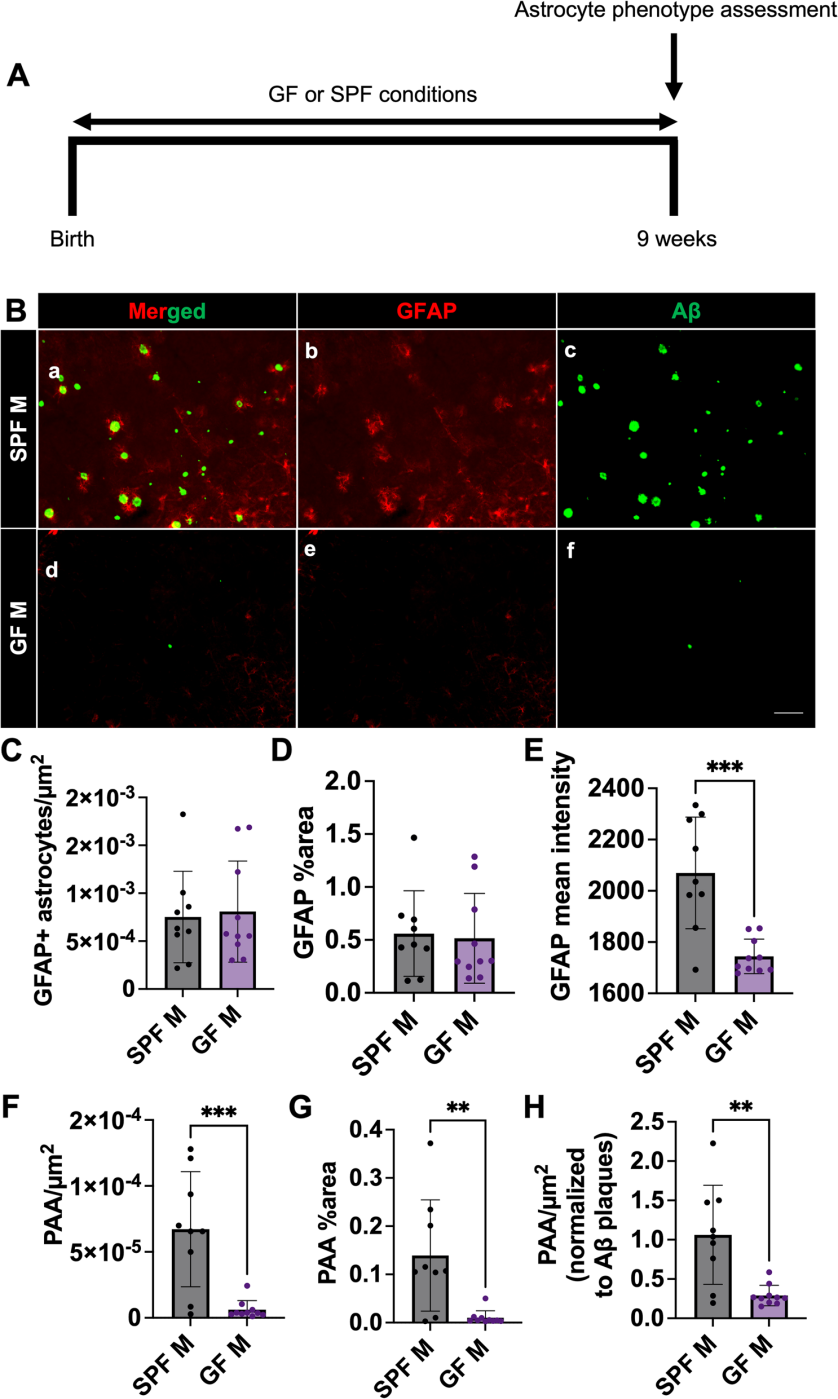
Germ-free environment results in a reduction in GFAP + astrocyte intensity and PAAs in male APPPS1-21 mice. (A) Experimental schematic. (B) Representative merged (a, d), GFAP (b, e) and Aβ (c, f) immunosignal images from specific pathogen free (SPF) raised males (a-c) and germ-free (GF) raised males (d-f). (C) Quantification of GFAP + astrocytes/µm^2^. (D) GFAP + astrocyte percent area. (E) GFAP mean intensity. (F) Plaque-associated astrocytes/µm^2^. (G) Plaque-associated astrocyte percent area. (H) Plaque-associated astrocytes/µm^2^ normalized to Aβ plaques/µm^2^ in SPF male and GF male APPPS1-21 mice. M = male. Data expressed as mean ± standard deviation. SPF M *N* = 9, GF M *N* = 10. Statistics calculated using two-tailed unpaired student’s t-tests. 4 sections used per animal. * denotes a *p*-value ≤ 0.05, ** indicates *p*-value ≤ 0.01, *** indicates *p*-value ≤ 0.001, and **** indicates a *p*-value of ≤ 0.0001. Scale bar indicates 100 µm

**Fig. 6:**
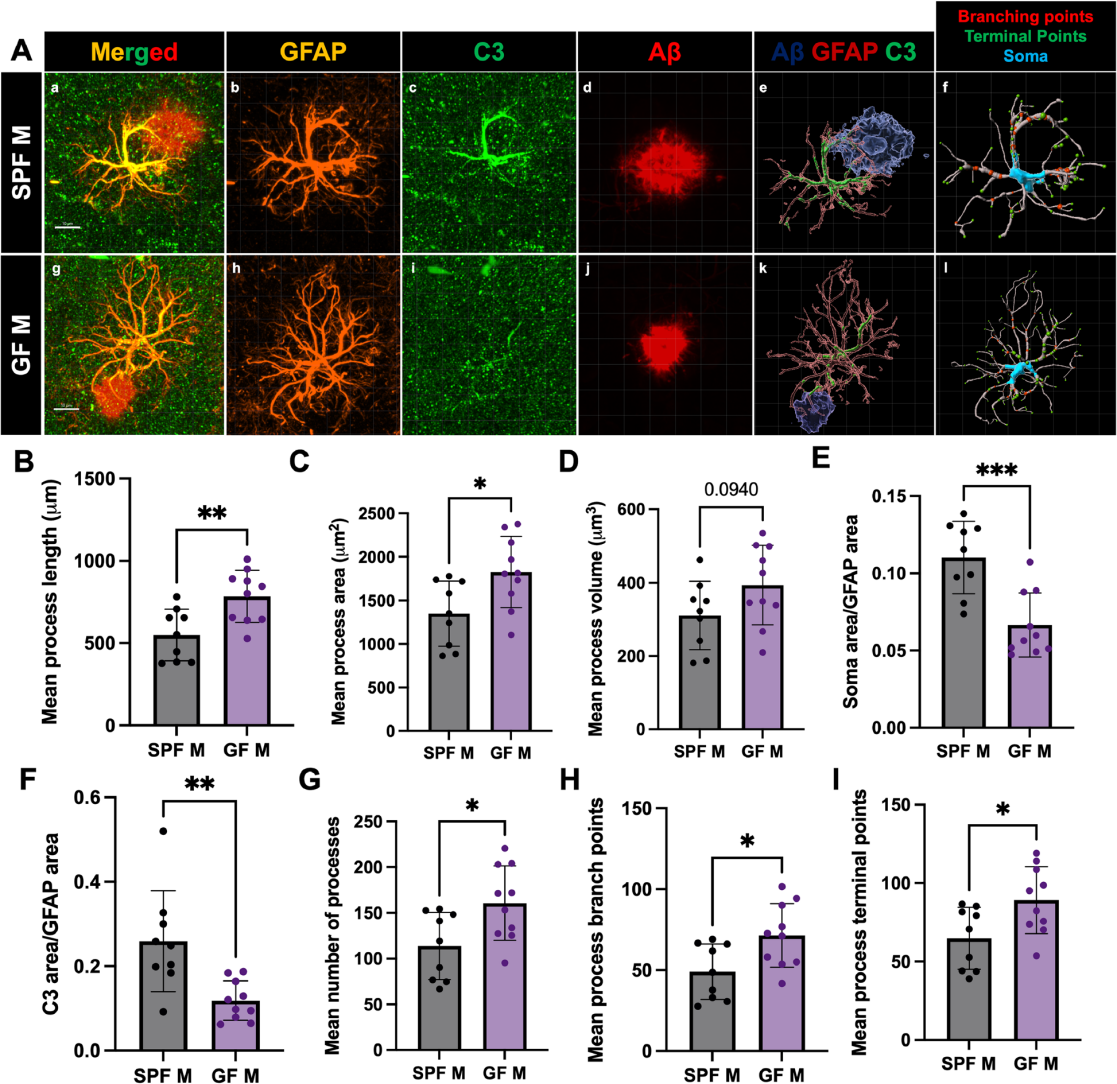
Germ-free environment alters astrocyte morphology and C3 area in a similar manner to short-term abx. (A) Representative GFAP, C3, and Aβ merged astrocyte z-stack maximum projections (a, g), IMARIS 3D reconstructions (e, k), and IMARIS filament 3D reconstructions (f, l) for SPF M (a-f) and GF M (g-l). GFAP (b, h), C3 (c, i), and Aβ (d, j) shown as separate channels from merged images. (B) Quantification and comparison of astrocyte mean process length sum (µm). (C) Astrocyte mean process area sum (µm^2^). (D) Astrocyte mean process volume sum (µm^3^). (E) Astrocyte soma area/GFAP area. (F) Astrocyte C3 area/GFAP area. (G) Astrocyte mean number of processes. (H) Astrocyte mean number of process branch points. (I) Astrocyte mean number of process terminal points between SPF male and GF male mice. M = male. Data expressed as mean ± standard deviation. SPF M *N* = 9, GF M *N* = 10. Statistics calculated using two-tailed unpaired student’s t-tests. 4 sections used per animal. * denotes a *p*-value ≤ 0.05, ** indicates *p*-value ≤ 0.01, *** indicates *p*-value ≤ 0.001, and **** indicates a *p*-value of ≤ 0.0001. Scale bars indicate 10 µm

**Fig. 7:**
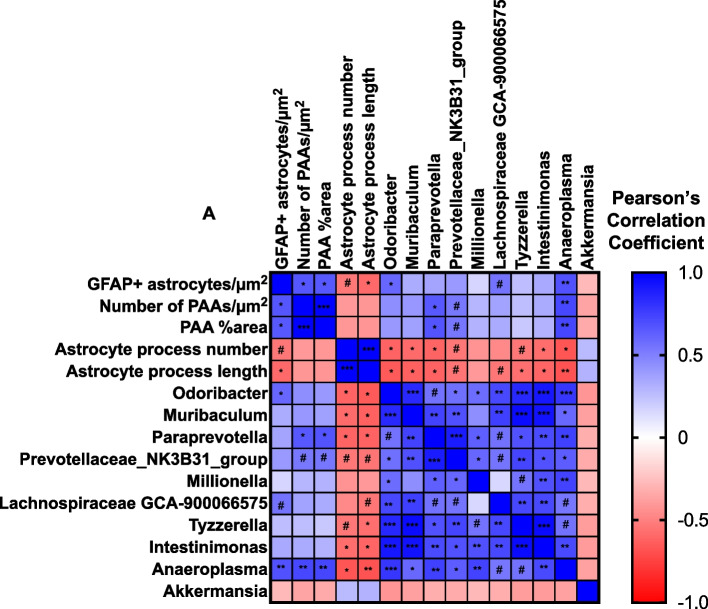
Astrocyte phenotypes correlate with bacterial genera altered by abx treatment. Correlational analysis between levels of GFAP + astrocytes/µm^2^, PAAs/µm.^2^, PAAs (%area), astrocyte process number, astrocyte process length, *Odoribacter, Muribaculum, Paraprevotella, Prevotellaceae, Millionella, Lachnospiraceae GCA-900066575, Tyzzerella,* and *Intestinimonas, Anaeroplasma, and Akkermansia.* Vehicle and abx-treated male APPPS1-21 mice are included in the analysis. Pearson’s R values are represented by colors where blue is a positive correlation and red is a negative correlation. *N* = 13 mice (6 VHL, 7 ABX). Two-tailed *p*-values and Pearson’s correlation coefficients were calculated for each comparison. A confidence interval of 95% was used. P and R values for each comparison are available in Additional file 12. * denotes a *p*-value ≤ 0.05, ** indicates *p*-value ≤ 0.01, *** indicates *p*-value ≤ 0.001. # denotes a *p*-value > 0.05 and ≤ 0.01

**Fig. 8:**
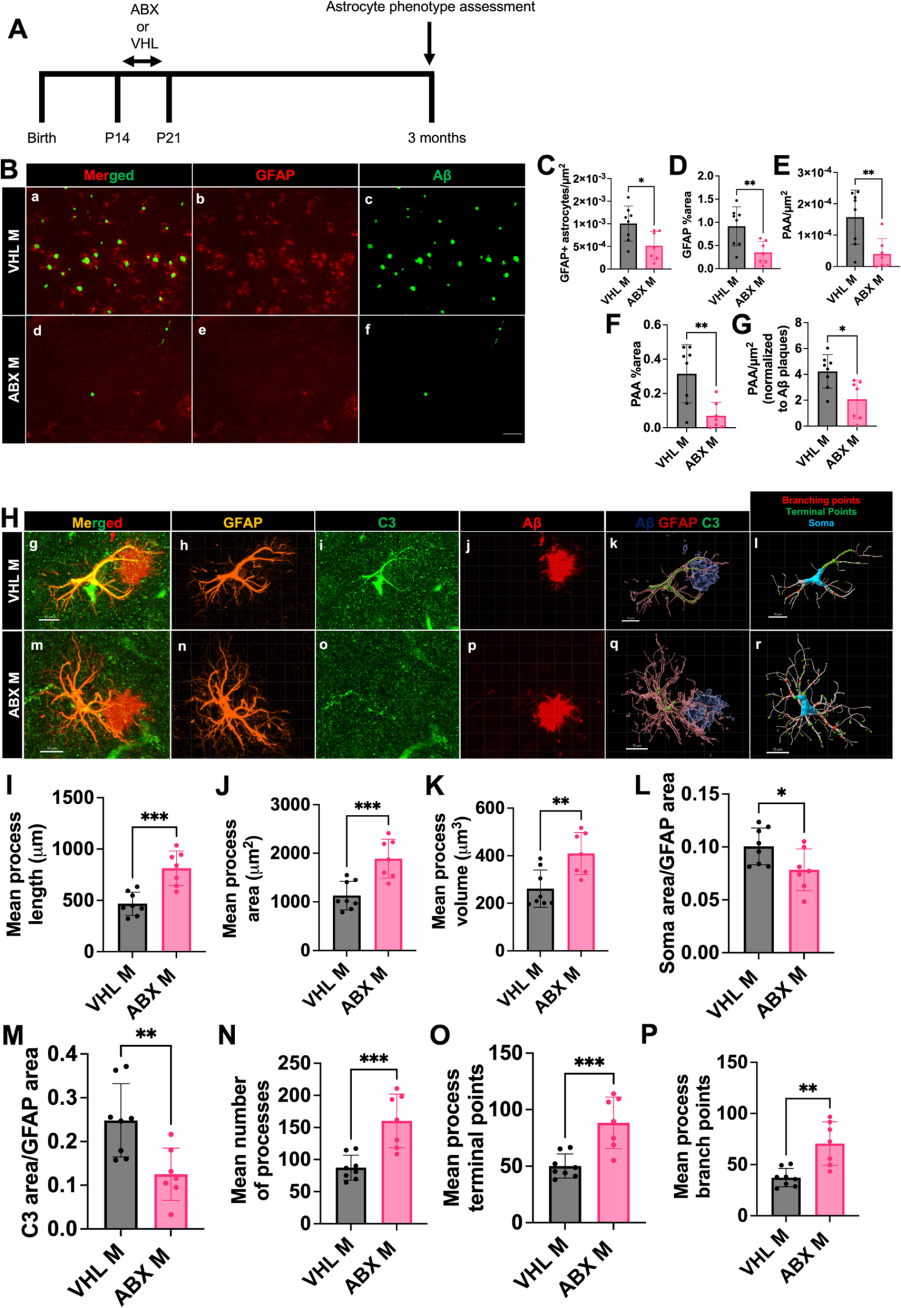
Short term antibiotics reduces GFAP + astrocytes, PAAs, astrocytic C3, and alters astrocyte morphology in APPPS1-21 mice at 3 months of age. (A) Experimental schematic. (B) Representative merged (a, d), GFAP (b, e) and Aβ (c, f) immunosignal images from VHL M (a-c) ABX M (d-f). (C) Quantification of GFAP + astrocytes/µm^2^. (D) GFAP + astrocyte percent area. (E) Plaque-associated astrocytes/µm^2^. (F) Plaque-associated astrocyte percent area. (G) Plaque-associated astrocytes/µm^2^ normalized to Aβ plaques/µm^2^ in VHL male and ABXF male APPPS1-21 mice. (H) Representative GFAP, C3, and Aβ merged astrocyte z-stack maximum projections (g, m), IMARIS 3D reconstructions (k, q), and IMARIS filament 3D reconstructions (l, r) for VHL male (g-l), ABX male (m-r) groups. GFAP (h, n), C3 (i, o), and Aβ (j, p) shown as separate channels from merged images. (I) Quantification and comparison of astrocyte mean process length sum (µm). (J) Astrocyte mean process area sum (µm^2^). (K) Astrocyte mean process volume sum (µm^3^). (L) Astrocyte soma area/GFAP area. (M) Astrocyte C3 area/GFAP area. (N) Astrocyte mean number of processes. (O) Astrocyte mean number of process branch points. (P) Astrocyte mean number of process terminal points between VHL male and ABX male groups. M = male. Data expressed as mean ± standard deviation. VHL M *N* = 8, ABX M *N* = 7. Statistics calculated using two-tailed unpaired student’s t-tests. 4 sections used per animal. * denotes a *p*-value ≤ 0.05, ** indicates *p*-value ≤ 0.01, *** indicates *p*-value ≤ 0.001, and **** indicates a *p*-value of ≤ 0.0001. Scale bars indicate 100 µm in B and 10 µm in H

**Fig. 9:**
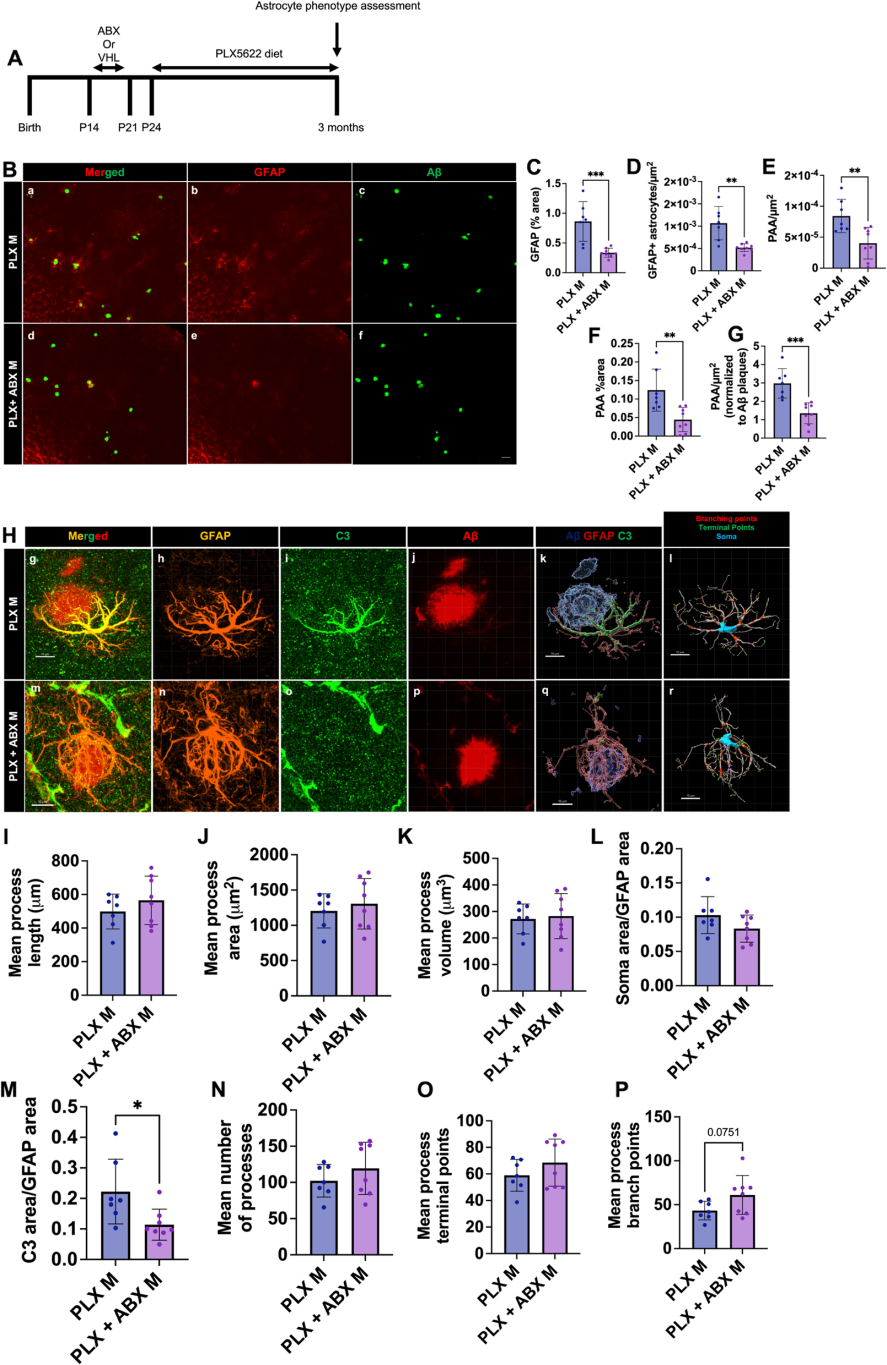
CSF1R inhibitor-mediated microglial depletion reveals microglial dependent and independent effects of abx on astrocyte phenotypes. (A) Experimental schematic. (B) Representative merged (a, d), GFAP (b, e) and Aβ (c, f) immunosignal images from VHL M (a-c) ABX M (d-f). (C) Quantification of GFAP + astrocyte number/µm^2^. (D) GFAP + astrocyte percent area. (E) Plaque-associated astrocyte number/µm^2^. (F) Plaque-associated astrocyte percent area. (G) Plaque-associated astrocytes/µm^2^ normalized to Aβ plaques/µm^2^ in PLX male and PLX + ABX male APPPS1-21 mice. (H) Representative GFAP, C3, and Aβ merged astrocyte z-stack maximum projections (g, m), IMARIS 3D reconstructions (k, q), and IMARIS filament 3D reconstructions (l, r) for PLX male (g-l), PLX + ABX male (m-r) groups. GFAP (h, n), C3 (i, o), and Aβ (j, p) shown as separate channels from merged images. (I) Quantification and comparison of astrocyte mean process length sum (µm). (J) Astrocyte mean process area sum (µm^2^). (K) Astrocyte mean process volume sum (µm^3^). (L) Astrocyte soma area/GFAP area. (M) Astrocyte C3 area/GFAP area. (N) Astrocyte mean number of processes. (O) Astrocyte mean number of process branch points. (P) Astrocyte mean number of process terminal points between PLX male and PLX + ABX male groups. M = male. Data expressed as mean ± standard deviation. PLX M *N* = 7, PLX + ABX M *N* = 8. Statistics calculated using two-tailed unpaired student’s t-tests. 4 sections used per animal. * denotes a *p*-value ≤ 0.05, ** indicates *p*-value ≤ 0.01, *** indicates *p*-value ≤ 0.001, and **** indicates a *p*-value of ≤ 0.0001. Scale bars indicate 50 µm in B and 10 µm in H

Statistical analysis was performed using GraphPad Prism 9 software for all studies except the microbiome profiling studies. Pairwise comparisons using the Mann–Whitney test were used for alpha diversity statistics (Supplemental Fig. 2D, E). Pairwise ANOSIM testing was used for beta diversity analysis statistics (Supplemental Fig. 2F). Two-tailed unpaired student’s t-tests were used for single group comparisons (Figs. 1, 2, 3, 4, 5, 6, 8 and 9 and Supplemental Fig. 1, 4, 7, 8, 9G-I). For comparison of genera between ABX and VHL groups, false discovery rate (FDR) adjusted *p*-value was used (Supplemental Fig. 2). Pearson’s correlation coefficients and two-tailed *p*-values were calculated using GraphPad Prism 9 (Fig. 7) with a confidence interval of 95%. A *p*-value of ≤ 0.05 was considered statistically significant throughout the study.

## Results

### Administration of short-term antibiotics alter cecum weight, gut microbiota profile, and Aβ amyloidosis in male and female APPPS1-21 mice at 9 weeks of age

In order to observe how astrocyte phenotypes change in response to GMB perturbation in the context of cerebral amyloidosis, we treated male and female APPPS1-21 mice from postnatal day 14 to postnatal day 21 with a broad-spectrum abx cocktail containing kanamycin, gentamicin, colistin, metronidazole, and vancomycin or water control as described previously (Fig. 1A) [27]. The day before sacrifice, at 9 weeks of age, fecal pellets were collected from all mice and after sacrifice cecum and body weights were recorded. As expected, we observed no difference in body weight between control and abx-treated groups in either male or female mice (Supplemental Fig. 1A, D). We observed a significant increase in cecum weight and cecum to body weight ratio in male and female abx-treated mice compared to control-treated mice (Supplemental Fig. 1B, C, E, F) as has been previously reported [24–27]. After confirming increases in cecum weight in abx-treated male and female mice, we sought to determine how abx treatment altered GMB profile in male and female APPPS1-21 mice. 16 s ribosomal RNA (rRNA) amplicon sequencing of mouse feces was used to profile the GMB in male and female mice. Principal component analysis (PCA) of the 16 s sequencing showed a separation of abx and control treated groups in both the male and female mice at the genus level (Supplemental Fig. 2A-C). Alpha diversity (Shannon index) analysis showed a significant decrease in abx treated male mice relative to control mice (*p*-value = 0.0082) (Supplemental Fig. 2D). However, alpha diversity was not changed in abx treated female mice relative to control female mice (*p*-value = 0.65) (Supplemental Fig. 2E). Beta-diversity analysis showed a significant separation of microbial diversity between the abx and control treated groups in both the male (pairwise ANOSIM, *p*-value = 0.002) and female (pairwise ANOSIM, *p*-value = 0.002) cohorts at the genus level (Supplemental Fig. 2F). We found several significant changes in both males and female at the genus level in abx-treated mice compared to control. In male mice treated with abx, we observed a significant increase in *Akkermansia* and a significant decrease *in Odoribacter, Muribaculum, Pararevotella, Prevotellaceae, Millionella, Lachnospiraceae GCA-900066575, Tyzzerella,* and *Intestinimonas, Anaeroplasma* (Supplemental Fig. 3, Additional file 11). In female mice, we observed a significant increase in *Paraprevotella and a* decrease in *Muribaculum, Millionella, Intestinimonas,* and *Anaeroplasma* (Supplemental Fig. 3, Additional file 11).

Previous studies have shown that short-term abx causes a reduction in Aβ plaque load in the brain of male APPPS1-21 mice [24–27]. To confirm this observation, immunohistochemistry (IHC), was performed to quantify Aβ in the cortex of APPPS1-21 male and female mice at 9 weeks of age. We observed the presence of Aβ in the cortex in both male and female mice at 9 weeks (Fig. 1B). As expected, abx-treated male mice had a significantly lower number of Aβ plaques/µm^2^ and Aβ plaque percent area compared with vehicle-treated male mice (Supplemental Fig. 4A, B). Consistent with previous reports, there was no difference in Aβ plaque burden in female mice treated with abx compared with vehicle-treated female mice (Supplemental Fig. 4D, E). These data, which are similar to previous studies using antibiotics in amyloidosis models [24–27], confirm that GMB composition modulates Aβ burden in a sex-specific manner. Additionally, we found that abx treatment decreases Aβ plaque size in both male and female mice (Supplemental Fig. 4C, F).

### Administration of short-term antibiotics reduces GFAP + astrocytes and plaque-associated astrocytes in the brain of male APPPS1-21 mice at 9 weeks of age

Microglia are likely important players in modulating GMB-induced control of Aβ plaque deposition [24–27, 41]. Additionally, microglia have been shown to interact with astrocytes through several different mechanisms that can cross regulate function and modulate disease pathology [11, 12, 55–59]. Therefore, we sought to study astrocyte phenotypes in the context of GMB perturbation. Glial fibrillary acidic protein (GFAP) is an intermediate filament protein that is expressed in astrocytes and is a widely used marker of reactive astrocytes [60]. GFAP levels were assessed using IHC in the cortex of 9-week-old male and female APPPS1-21 mice that were treated with abx or vehicle control. We observed a significant reduction in the number of GFAP + astrocytes/µm^2^ and GFAP + astrocyte percent area in abx-treated male mice compared with vehicle-treated control mice (Fig. 1C, D). However, we observed no significant differences in GFAP + astrocytes in abx-treated female mice compared with vehicle-treated controls (Fig. 1E, F). Because GFAP + astrocytosis was reduced in abx-treated mice, we were also interested in whether astrocytic recruitment to Aβ plaques was also reduced. We therefore compared the number of plaque-associated GFAP + astrocytes (PAA) between vehicle and abx-treated mice. There was a decrease in the number of PAAs/µm^2^ and PAA percent area in the abx-treated male mice (Fig. 1G, H), but we did not observe any differences in PAAs between abx- and vehicle-treated female mice (Fig. 1J, K). We sought to determine whether the abx-mediated reduction in PAAs was due to the reduction in Aβ plaques. When we normalized PAAs/µm^2^ to Aβ plaques/µm^2^, there was still a reduction in PAAs, suggesting that abx treatment indeed reduces GFAP+ reactive astrocyte recruitment to Aβ plaques (Fig. 1I). To validate our IHC data, we next performed immunoblotting using hemicortex lysates (Fig. 1M-T, Supplemental Fig. 5) from abx and vehicle treated mice. Consistently, we found a significant reduction in GFAP protein in abx treated male mice compared to vehicle controls (Fig. 1M, N, Supplemental Figs. 5–6) but found no difference in females (Fig. 1Q, R, full blots in Supplemental Figs. 5–6). Additionally, because previous studies indicate abx reduces microglial activation in APPPS1-21 male mice, we also immunoblotted for ionized calcium-binding adaptor molecule 1 (IBA1) (Fig. 1M, Q). We found no significant change in IBA1 protein levels in abx treated males (Fig. 1O) or females (Fig. 1S) compared to vehicle controls. Furthermore, although GFAP and IBA1 protein levels were weakly positively correlated in male (Fig. 1P) and female animals (Fig. 1T), these correlations did not reach statistical significance, suggesting that abx-mediated GFAP protein changes may happen independently of microglia.

Finally, we assessed how other populations of astrocytes aside from GFAP + astrocytes may be altered by abx treatment. S100B has been shown to be enriched in protoplasmic gray matter astrocytes and may represent a population of astrocytes distinct from GFAP + cortical astrocytes [61]. To ensure S100B + cells were astrocytes and not oligodendrocytes which also express S100B [61], co-labeling with Sox9 an astrocyte specific nuclear marker [62] was performed in male abx and vehicle control treated APPPS1-21 tissue. However, we found that there was no difference in S100B + cells, Sox9 + cells, or S100B + Sox9 + cells (Supplemental Fig. 7). These results suggest that the GMB does not modulate S100B + astrocyte induction and is more important in GFAP + reactive astrocyte induction.

Taken together, our results indicate that abx-mediated GMB alteration results in a sex-specific decrease in GFAP + reactive astrocytosis and association of reactive astrocytes with Aβ plaques, suggesting that GMB composition plays an important role in GFAP + reactive astrocytosis induction and recruitment to Aβ plaques.

### Administration of short-term antibiotics alters astrocyte morphology and C3 + astrocyte reactivity in the brain of male APPPS1-21 mice

Astrocytes change their morphology and upregulate complement component *C3* expression in the context of inflammatory stimuli, including Aβ deposits [11, 17, 18, 58, 60, 63]. C3 has been shown to mark a subtype of neurotoxic reactive astrocytes which are abundantly present in the brains of patients with various neurodegenerative diseases, including AD [11]. Additionally, astrocytic C3 has been shown to compromise neuronal function and microglial Aβ phagocytosis [58, 64] leading to increased Aβ plaques and decline in cognitive function. In order to more deeply understand astrocyte phenotypes in the context of GMB perturbations, we assessed PAA morphology and C3 + reactivity in male and female abx or vehicle-treated mice. To assess astrocyte morphology, we performed IHC with antibodies specific for GFAP, C3, and Aβ and captured 3D confocal z-stacks of PAA in the mouse cortex (Fig. 2A). IMARIS software was used to generate 3D reconstructions of confocal images and quantify morphological parameters and C3 signals (Fig. 2A, Supplemental Fig. 8). We observed significant morphological changes in PAA from male abx-treated mice compared with vehicle-treated controls. Specifically, astrocytes from abx-treated male mice had increased process length (Fig. 2B), area (Fig. 2C), and volume (Fig. 2D), and an increased number of processes (Fig. 2G), branch points (Fig. 2H), and terminal points (Fig. 2I) compared with PAAs in vehicle-treated male mice. Furthermore, astrocytes from abx-treated male mice had a reduction in soma area/GFAP area (Fig. 2E) and C3/GFAP signal (Fig. 2F) compared with vehicle-treated male mice, suggesting that astrocytes in abx-treated animals are less reactive in response to plaques than astrocytes in vehicle-treated animals. Because several previous studies have found that ezrin is a major regulator of astrocyte morphology, is decreased in APP/PS1 mice, and that deficiency of ezrin is associated with an increased GFAP + reactive astrocyte phenotype, we assessed ezrin levels via immunoblotting (Fig. 2J) [20, 65, 66]. We found a significant increase in ezrin in abx treated mice compared to vehicle controls (Fig. 2K). Interestingly, protein levels of ezrin strongly positively correlated with number of astrocyte processes (Fig. 2M), branch points (Fig. 2N), terminal points (Fig. 2O), and process lengths (Fig. 2P). Ezrin levels also negatively correlated with GFAP + astrocytes/µm^2^ (Fig. 2Q) and C3 levels (Fig. 2R), similar to previous reports that ezrin deficiency increases GFAP + reactive astrocytosis. Additionally, we also assessed brain IL-33 levels (Fig. 2J). IL-33 is an anti-inflammatory cytokine, which is expressed in the brain primarily by astrocytes. IL-33 has been shown to be neuroprotective in models of stroke and brain infection, regulate microglial phagocytosis, reduce amyloid plaques, and increase excitatory synaptic transmission [67–71]. We found that IL-33 is increased in the brains of in abx treated mice compared to vehicle controls (Fig. 2L), suggesting that astrocytes in abx-treated mice take on an anti-inflammatory phenotype and increase their release of IL-33. Although changes in astrocyte morphology and C3 signal were seen in response to abx-mediated GMB perturbation in male mice, there were no differences in these parameters in female abx-treated mice compared with respective controls (Supplemental Fig. 8). Taken together, these data suggest that when GMB composition is altered by abx in male mice, astrocytes change their morphology and suppress their C3-associated reactive program and take on a more homeostatic, anti-inflammatory phenotype in response to Aβ plaques.

### Fecal matter transplant restores GFAP + astrocytes and PAA in the brains of male APPPS1-21 mice

It is possible that in addition to altering GMB composition, abx treatment could cause off-target effects. Therefore, we asked whether abx-mediated alterations of the GMB is directly related to changes in astrocytes. In order to assess this, we treated male mice from P14-21 with broad spectrum abx and then from P24 until sacrifice administered either water vehicle or performed fecal matter transplant (FMT) from untreated age-matched donor mice to restore GMB composition (Fig. 3A). We performed IHC to label GFAP and Aβ in brain tissue sections from abx and abx + FMT treated APPPS1-21 male mice (Fig. 3A, B). Quantification of the number of Aβ plaques/µm^2^ and Aβ plaques percent area showed an increase in the abx + FMT group compared to abx alone, suggesting that restoring the GMB increased amyloidosis, as has been previously reported [27] (Supplemental Fig. 4G, H). Additionally, the Aβ plaque size was trending to increase in the abx + FMT group compared to the abx only treated group (*p* = 0.07) (Supplemental Fig. 4I). We then compared the number of GFAP + astrocytes/µm^2^ and GFAP + astrocyte percent area between abx-treated male mice and abx + FMT-treated male mice. We observed an increase in GFAP + astrocytes in the abx + FMT group (Fig. 3C, D), suggesting that restoring the GMB restored GFAP + astrocytosis. Additionally, we quantified PAAs and found that FMT-treated male mice had an increased number of PAA/µm^2^ and PAA percent area compared with abx-treated mice (Fig. 3E, F). We also found that this increase was still present when PAA/µm^2^ was normalized to Aβ plaques/µm^2^ (Fig. 3G). Together, these data suggest that restoring the GMB in abx-treated mice results in a restoration of GFAP + astrocytosis and PAAs, and that the GMB directly modulates GFAP + astrocytosis and astrocytic recruitment to Aβ plaques.

### Fecal matter transplant restores astrocyte morphology and C3 + astrocyte reactivity in the brain of male APPPS1-21 mice

We next compared astrocyte morphology and C3 reactivity in abx-treated or abx + FMT treated mice (Fig. 4A). Compared to abx-treated male APPPPS1-21 mice, abx + FMT treated had significant changes in PAA morphology consistent with the phenotype of vehicle control treated APPPS1-21 male mice (Fig. 2). Astrocytes from abx + FMT treated male mice had decreased process lengths (Fig. 4B), areas (Fig. 4C), volume (Fig. 4D), cell body size (Fig. 4E) and a decreased number of processes (Fig. 4G), branch points (Fig. 4H), and terminal points (Fig. 4I) compared with abx-treated male mice. Additionally, we observed an increase in astrocytic C3 signal (Fig. 4F) in abx + FMT treated mice compared with abx-treated male mice. These results suggest that FMT from donor APPPS1-21 mice restores astrocyte morphology and C3 expression back to control levels. These results indicate that GMB perturbations directly modulate astrocyte morphology and astrocyte-specific C3 expression.

### Abx-mediated alterations in astrocyte-associated transcripts

Recently, Jiwaji et al. (2022) [52] explored astrocyte-specific transcriptomic changes in amyloid and tau models of AD. Furthermore, they used previously published astrocyte-associated RNAseq data to establish LPS [53], MCAO [53], pan-reactive [53], and aging [54] astrocyte gene modules. These gene modules contain genes which are differentially expressed in astrocytes in stimulus-induced groups compared to control. For the LPS, MCAO, and pan-reactive gene lists, they reanalyzed data from Zamanian et al. (2012) [53] to curate larger gene modules with clear ranking criteria. LPS and MCAO-induced genes had to be ranked in the top 100 genes for the respective stimulus and at least 50 places lower in the other stimulus. For the pan-reactive list, the gene had to be in the top 250 in both LPS and MCAO list with no more than 50 positions difference in ranking in the two stimulation paradigms. Jiwaji et al. (2022) [52] used these gene modules to determine whether amyloid and tau induced transcriptomic changes in astrocytes are similar to acute injury and aging induced transcriptomic changes in astrocytes. To determine how abx-mediated GMB alteration might change astrocyte-related gene transcripts, we assessed changes in amyloid (Supplemental Fig. 9A), LPS (Supplemental Fig. 9B), MCAO (Supplemental Fig. 9C), pan-reactive (Supplemental Fig. 9D) and aging gene modules (Supplemental Fig. 9E, F) in abx-treated male mouse vs vehicle-treated male mouse cortex from bulk RNAseq data reported by Dodiya et al. (2022) [27]. We found that while the majority of the amyloid (> 1 fold change) (Supplemental Fig. 9A) and LPS induced genes (Supplemental Fig. 7B) in astrocytes were decreased in abx-treated male mouse cortex compared with vehicle-treated mice, only some were decreased to a statistically significant degree. Furthermore, we observed that while the majority of MCAO-induced (Supplemental Fig. 9C) and aging-reduced (Supplemental Fig. 9F) astrocyte genes were increased in abx-treated male mouse cortex compared with vehicle-treated mice, only a few showed significant changes. We did not observe significant differences in the proportion of genes that were up- or down-regulated in the pan-reactive (Supplemental Fig. 9D) or aging-induced (Supplemental Fig. 9E) astrocyte gene modules. However, roughly half of the genes were decreased, implying that there was a partial decrease in pan-reactive and aging-induced astrocyte genes. Some particularly interesting genes that were significantly altered in the Dodiya et al. (2022) RNAseq dataset were *H2-d1* (downregulated), *Emp1* (upregulated), and *Cxcl10* (downregulated). *H2-d1* is a canonical pro-inflammatory “A1” astrocyte subtype marker and *Emp1* is a neurotrophic, neuroprotective “A2” astrocyte subtype marker [11, 53, 54]. *Cxcl10* is a “pan-reactive” astrocyte marker [11, 53, 54] and has been shown to be highly expressed in a subtype of neuroinflammatory astrocytes [17]. Additionally, astrocyte-derived CXCL10 binds to CXCR3 on T-cells to drive T cell-mediated neuroinflammation. Disruption of this axis could play a role in GMB-mediated control of AD pathogenesis [72, 73]. We verified *H2-d1*, *Emp1*, and *Cxcl10* gene changes by using qPCR with primers against these genes (Supplemental Fig. 9G-I). Overall, these gene module expression changes may suggest that when the GMB is perturbed by abx treatment, astrocytes downregulate genes important for responding to amyloid and inflammatory stimuli like LPS and gain a neuroprotective response, as seen in response to MCAO [53, 74]. However, it should be noted that many of the genes represented in these gene modules are not exclusively expressed in astrocytes, so future astrocyte-specific transcriptomic studies will need to be performed to confirm GMB-induced astrocyte-specific gene expression changes. Additionally, the amyloid model chosen in Jiwaji et al. (2022) was the APPswe/PS1dE9 mouse model [75], which has the same APP Swedish mutation (KM670/671NL) as the APPPS1-21 mouse, but the presenilin-1 mutation is delta E9 rather than L166P as in the APPPS1-21 mouse.

### Germ-free environment results in a reduction in astrocytic GFAP intensity and PAAs in male APPPS1-21 mice

While short-term treatment with broad-spectrum abx alters the GMB and reduces bacterial diversity (Supplemental Fig. 2D), we asked whether the absence of the GMB would have a similar effect on astrocyte phenotypes compared with the short-term abx treatment regimen. We performed IHC using anti-Aβ and anti-GFAP antibodies of tissue sections from 9-week-old APPPS1-21 male mice that were raised in a conventional specific pathogen free (SPF) or germ-free (GF) environment (Fig. 5A, B). Mice raised in the GF environment do not develop a GMB [76]. We observed a significant reduction in the number of Aβ plaques/µm^2^ and Aβ plaque percent area in the cortex of GF mice compared with SPF mice (Supplemental Fig. 4J, K), as was previously reported [34]. We also found a reduction in Aβ plaque size in GF housed mice compared to SPF housed mice, similar to abx-treated mice (Supplemental Fig. 4L). In contrast to our results in short-term abx treated mice, we observed no differences in the overall number of GFAP + astrocytes/µm^2^ or GFAP + astrocyte percent area in GF cortex compared with SPF mouse cortex (Fig. 5C, D). However, we did observe an overall decrease in the mean intensity of GFAP signal in GF mouse cortex compared to SPF, suggesting there is a reduction of GFAP expression in astrocytes in GF mice compared to SPF mice (Fig. 5E). We also quantified the number of PAA/µm^2^ (Fig. 5F) and PAA percent area (Fig. 5G) and observed significant reductions in GF mouse cortex compared with SPF mouse cortex. We also found that this reduction in PAA/µm^2^ was maintained when normalized to Aβ plaques/µm^2^ (Fig. 5H). Taken together, these data indicate that APPPS1-21 male mice raised in GF conditions, devoid of a GMB, have similar numbers of GFAP + astrocytes compared to SPF conditions, but the astrocytes in GF mice have low levels of GFAP expression and are less frequently recruited to amyloid plaques similar to what is observed in short-term abx-treated mice.

### Germ-free environment alters astrocyte morphology and C3 area in a similar manner to short-term abx

In addition to studying the effect of GF environment on GFAP + astrocytosis and PAAs, we asked whether GF would alter PAA astrocyte morphology and astrocytic C3 levels. We performed IHC of brain sections from male GF and SPF animals with antibodies specific for Aβ, GFAP, and C3, acquired confocal z-stacks, and analyzed astrocyte morphology using IMARIS (Fig. 6A). In comparison with astrocytes from SPF animals, astrocytes from GF animals had increased process lengths (Fig. 6B), areas (Fig. 6C), and volumes (a non-significant trend) (Fig. 6D), and an increased number of processes (Fig. 6G), branch points (Fig. 6H), and terminal points (Fig. 6I). Additionally, we observed a reduction in C3/GFAP signals (Fig. 6F) in GF mice compared with SPF mice. Importantly, these changes in astrocytes from GF mice were similar to those observed in short-term abx-treated animals. These results suggest that a decrease in bacterial diversity in the GMB (via abx treatment) or in GF animals with a complete absence of the GMB results in similar astrocytic morphological changes and reductions in C3 + reactivity in the context of amyloidosis.

### Astrocyte phenotypes correlate with bacterial genera altered by abx treatment

Because we observed reductions in GFAP + astrocytosis, PAAs, and alterations in plaque associated astrocyte morphology upon abx treatment in male APPPS1-21 mice and had observed significant alterations in particular bacterial genera, we asked whether the astrocyte phenotypes correlated with normalized bacterial genera levels (Fig. 7A). We generated a Pearson’s correlation matrix with 9-week-old vehicle and abx treated male APPPS1-21 mouse levels of GFAP + astrocytes/µm^2^, PAAs/µm^2^, PAA percent area, astrocyte process number, astrocyte process length, *Odoribacter, Muribaculum, Paraprevotella, Prevotellaceae, Millionella, Lachnospiraceae GCA-900066575, Tyzzerella, Intestinimonas, Anaeroplasma, and Akkermansia* (Fig. 7A)*.* We observed significant correlations and several trends between astrocyte phenotypes and bacterial genera load (Fig. 7A). There were significant positive correlations between *Odoribacter* and *Anaeroplasma* levels and GFAP + astrocytes/µm^2^ and there was a positive correlative trend between *Lachnospiraceae GCA-900066575* and GFAP + astrocytes/µm^2^. *Anaeroplasma* and *Paraprevotella* significantly positively correlated with PAAs/µm^2^ and PAA percent area, while *Prevotellaceae* had a positive correlative trend with PAAs/µm^2^ and PAA percent area. These genera were all significantly decreased after abx treatment (Supplemental Fig. 3) and the presence of a positive correlation with these astrocyte phenotypes suggests that these bacteria may be important in mediating GFAP + reactive astrocytosis and astrocytic recruitment to Aβ pathology. Additionally, there were significant negative correlations between *Odoribacter, Muribaculum, Pararevotella, Intestinimonas, and Anaeroplasma* and astrocyte process number and negative correlative trends with *Prevotellaceae and Tyzzerella* (Fig. 7A). *Odoribacter, Muribaculum, Paraprevotella, Intestinimonas, Tyzzerella, and Anaeroplasma* significantly negatively correlated with astrocyte process length and *Prevotellaceae* and *Lachnospiraceae GCA-900066575 had negative* correlative trends with astrocyte process length. Importantly, this suggests that these bacteria may mediate reactive astrocyte morphological phenotype characterized by low number and length of processes. When these bacteria are depleted by abx, astrocytes shift into a more homeostatic morphologic phenotype with increased number and length of processes. Overall, these data suggest that abx-altered bacterial genera correlate with changes in astrocyte phenotypes, suggesting that these bacteria may be particularly important for regulating astrocytic response to Aβ plaque pathology.

### ABX reduces GFAP + astrocytes, PAAs, astrocytic C3, and alters astrocyte morphology in male APPPS1-21 mice at 3 months of age

Given that Aβ plaque deposition starts at 6 weeks of age in the APPPS1-21 mouse model, our studies in mice 9 weeks of age are relatively early in amyloidosis. Thus, we asked whether GMB-mediated astrocyte changes persisted when there was more amyloid in the brain microenvironment. To answer this question, we assessed cortical GFAP + astrocytosis, PAA, and Aβ amyloidosis levels by using IHC in 3-month-old APPPS1-21 mice that had been treated with vehicle or abx from P14-21 (Fig. 8A, B). While the 3 month timepoint is still relatively early in amyloidosis, there is significantly more amyloid in the cortical microenvironment compared to 9 weeks, and it has been shown that abx-mediated changes in amyloid and microglial phenotypes are sustained at 3 months [27]. We confirmed abx treatment resulted in a reduction in Aβ plaques/µm^2^, Aβ plaque percent area, and Aβ plaque size at the 3-month time point (Supplemental Fig. 4J-L). Importantly, we found that abx treatment results in a reduction in GFAP + astrocytes/µm^2^ and GFAP percent area compared to vehicle control (Fig. 8C, D) at the 3-month timepoint. Additionally, we observed a reduction in PAA/µm^2^ and PAA percent area (Fig. 8E, F). Confirming that this decrease is not just due to the overall decrease in amyloid plaques due to abx treatment, we also observed an abx-mediated decrease in PAA/µm^2^ when this measure was normalized to Aβ plaques/µm^2^ (Fig. 8G). We also were interested in whether abx-induced morphological and C3 changes persisted at the 3-month time point. Therefore, we used IHC to label GFAP, C3, and Aβ plaques, performed confocal imaging of PAAs and quantified morphological parameters and C3 co-labeling with GFAP between abx-treated and vehicle treated APPPS1-21 male mice (Fig. 8H). Similar to the 9 weeks timepoint, abx treatment leads to an increase in mean process length (Fig. 8I), area (Fig. 8J), volume (Fig. 8K), processes (Fig. 8N), terminal points (Fig. 8O), and branch points (Fig. 8P) at the 3 month timepoint. We also found that abx leads to a decrease in astrocytic C3 expression (Fig. 8M) and soma size (Fig. 8L) at 3 months consistent with the 9-week timepoint.

### GMB mediated regulation of astrocyte phenotypes is both microglial dependent and independent

It is well known that microglia and astrocytes communicate and previous studies have found that the GMB regulates microglial phenotype [24–27, 34, 41, 42, 77, 78], so we wanted to assess if GMB-mediated control of astrocyte phenotypes was dependent or independent of microglia. Here, we utilized brain sections previously characterized from Dodiya et al. 2022 [27] from 3-month-old APPPS1-21 mice treated with ABX or vehicle control from P14-21 and then treated with CSF1R inhibitor diet (PLX5622) from P24 until 3 months (Fig. 9A). CSF1R inhibition depletes microglia and other CSF1R myeloid cells from the brain [79], and Dodiya et al. 2022 had previously confirmed microglial depletion in the sections used [27]. We chose to look at the 3-month timepoint because Dodiya et al. 2022 [27] found that PLX-mediated microglial depletion nearly eliminated amyloid plaques when mice were aged until 9 weeks, but did not affect amyloid plaque load when the mice were aged until 3 months. In order to study how abx alters amyloid induced astrocyte phenotypes in the absence of microglia we evaluated the 3 month timepoint. Using IHC to stain for GFAP and Aβ (Fig. 9B), we first confirmed that there was no change in Aβ plaques/µm^2^, Aβ plaque percent area, or Aβ plaque size between the PLX and the PLX + abx groups, as previously reported [27] (Supplemental Fig. 4P-R). Interestingly, we found that in the absence of microglia, abx still reduced GFAP + astrocytes/µm^2^ (Fig. 9C), GFAP percent area (Fig. 9D), PAA/µm^2^ (Fig. 9E), and PAA percent area (Fig. 9F), suggesting that the GMB can regulate GFAP + reactive astrocyte induction and astrocytic recruitment to Aβ plaques independently of microglia. We then asked whether abx-induced astrocyte morphological and C3 expression changes are dependent on microglia. Therefore, we again used IHC to assess morphological features and C3 expression (Fig. 9H). We found no difference between mean process length (Fig. 9I), area (Fig. 9J), volume (Fig. 9K), processes (Fig. 9N), terminal points (Fig. 9O), branch points (Fig. 9P), or soma size (Fig. 9L) between abx and vehicle treated mice in the absence of microglia. However, we did find that abx still reduces astrocytic C3 expression even in the absence of microglia (Fig. 9M). These results indicate that GMB-mediated regulation of astrocyte morphology is likely dependent or partially dependent on microglia, but GMB control of astrocytic C3 expression (Fig. 9M) is independent of microglia. Together, our data suggests that abx-mediated changes in astrocyte phenotypes take place through both microglial independent and dependent mechanisms.

## Discussion

The impact of our study centers on several novel observations. For the first time, we report that the GMB regulates reactive astrocyte phenotypes in the context of amyloidosis. Although there have been previous studies linking the GMB with astrocytes in other disease models and contexts [22, 42, 56, 80], we are not aware of any studies that robustly link the GMB to astrocyte phenotypes in the context of amyloidosis. Our data reveal that the GMB regulates GFAP + reactive astrocytosis and astrocyte recruitment to amyloid plaques. Furthermore, our data show that astrocyte morphological phenotypes are altered by the GMB. Specifically, PAAs in 9-week-old and 3-month-old APPPS1-21 male mice treated with abx have a reduction in cell body size and an increased number and length of astrocyte processes. Importantly, 9-week-old APPPS1-21 male mice treated with abx and given FMT from donor untreated 9-week-old APPPS1-21 male mice restored cell body size and number and length of astrocyte processes to control levels, suggesting that the observed astrocyte changes are indeed a result of GMB manipulation. Additionally, FMT treatment restored the number of GFAP + astrocytes and the number of PAAs.

Our results indicate that abx-mediated changes in astrocytes are sex-specific as has been reported for amyloid and microglial phenotypes [26, 27]. It is possible that a higher dose of abx is needed to achieve the same effect on astrocytes and other brain phenotypes in females as males. This is evidenced by the fact that the same dose of abx in females alters substantially less bacterial genera (5 genera) than in males (10 genera). Future studies will need to test whether a higher dose of abx is sufficient to induce changes in astrocytes in female mice. Other possible reasons for sex-specific phenotypes after abx treatment could be due to differences in GMB-hormone interactions or sex-specific differences in astrocyte function [81]. Estrogen signaling has been shown to be important in astrocyte activation [82]. Furthermore, the GMB has been shown to regulate levels of testosterone, which has immunomodulatory effects [83]. Moreover, the mechanism by which GMB perturbation may alter astrocyte activity may be through short-chain fatty acid effects on astrocyte gene expression and phenotype. A recent study demonstrated that short-chain fatty acids can have sex-specific effects on astrocyte gene expression in vitro [80].

The GMB also regulates C3 + reactivity and cell morphology in PAAs. C3 has been previously shown to mark neurotoxic reactive astrocytes, which often localize to areas of neurodegeneration and are present in several neurodegenerative diseases [11]. Astrocytic C3 has also been shown to impair neuronal function and microglial Aβ phagocytosis [58, 64]. Our data shows that astrocytes in 9-week-old and 3-month-old APPPS1-21 male mice that were treated with abx postnatally have a reduction in C3 expression in PAAs. We also show an association between particular astrocyte morphological changes and GFAP/C3 + reactivity in 9 weeks and 3-month-old male APPPS1-21 mice that were treated with abx postnatally. Our results suggest that an increase in astrocyte process number and process length are associated with lower GFAP/C3 + reactivity in male APPPS1-21 mice. The process number and process length are also associated with increased ezrin levels, which is a regulator of astrocyte morphology. Our results are consistent with a recent study showing that APP/PS1 mice have reduced astrocyte territory size and ezrin expression associated with their reactive program compared to WT mice [20]. Our morphology data suggests that abx-treatment shifts astrocytes back into a more homeostatic, less reactive state. We also found an increase in IL-33 in abx treated mice compared to controls, which is an anti-inflammatory cytokine that is primarily produced by astrocytes in the brain. IL-33 promotes homeostatic synaptic plasticity, reduces amyloid plaque deposition, increases microglial phagocytosis of amyloid, improves amyloid-induced behavioral deficits, and is neuroprotective in stroke models [68, 70, 71]. Similar to our morphologic data, astrocyte signal transducer and activator of transcription 3 (Stat3) knockout mice [84] crossed to APP/PS1 mice, have reduced reactive astrocytosis and increased number of processes and length of processes, suggesting more processes are associated with a more homeostatic program. Our results are also similar to astrocytic morphological changes observed in abx treated P301S tau transgenic mice compared to vehicle controls [42]. It is possible that the specific astrocyte morphological changes identified in our study may be unique to this particular mouse line and age as this has not been tested in other amyloid mouse models. It is well accepted that there is remarkable astrocyte phenotype heterogeneity with respect to age, treatment, and sex in mouse models [17, 60, 85–87]. Our results in abx-treated male APPPS1-21 mice may represent a unique subset of astrocytes.

We found that complete absence of the GMB results in similar astrocyte changes as seen in short-term abx-treated male mice. GF mice have similar levels of GFAP + astrocytes in their brains compared with SPF mice treated with abx but have overall reduced GFAP expression. Additionally, there is a striking decrease of PAAs in GF male APPPS1-21 mice compared to SPF housed mice, suggesting that the GMB regulates reactive astrocytic recruitment to Aβ plaques. Furthermore, we observed a similar reduction in cell body size, increased number and length of astrocyte processes, and decreased C3 expression in PAAs in GF mice compared with SPF mice treated with abx. These data confirm our findings using short-term abx to alter the GMB and demonstrate the presence of a GMB-amyloid-astrocyte axis in AD model mice.

Correlational analysis shows that several bacterial genera which are depleted by abx correlate positively with GFAP + astrocytes/µm^2^, PAAs/µm^2^, and PAA percent area, suggesting that these microbes regulate GFAP + reactivity and recruitment to Aβ plaques. Furthermore, these same microbes negatively correlate with number of PAA processes and process length, suggesting that the abx-mediated increase in astrocyte processes and length is due to reductions in these bacteria. The microbes which most strongly correlated with pro-inflammatory astrocyte phenotypes were *Anaeroplasma*, *Paraprevotella*, and *Odoribacter*. Interestingly, these bacteria have been previously shown to be increased in mouse models of amyloidosis and tauopathy [37, 42, 88–92]. Our data raises the question of whether initial astrocyte reaction to amyloid pathology is potentially regulated by something other than just amyloid pathology itself, such as the GMB and peripheral immunity. This is in line with previous studies, which have found that GFAP + reactive astrocytes in the brain parenchyma do not correlate well with amyloid plaques in human AD [93]. Our results suggest that GMB composition may indeed regulate and modify astrocyte responses to Aβ plaques.

Based on previous reports that microglia can regulate astrocyte activation, we wanted to assess whether GMB manipulation effects on astrocytes are microglial dependent or independent. Using CSF1R inhibition to deplete microglia and other CSF1R + myeloid cells in vehicle and abx treated male APPPS1-21 mice, we found that abx-mediated reduction in GFAP + reactive astrocytes, PAAs, and astrocytic C3 expression was microglial independent. However, abx-mediated changes in astrocyte morphology were at least partially microglial dependent. Microglial independent GMB-mediated changes in astrocyte phenotype suggest that either the GMB directly affects astrocyte phenotype, or these phenotypes may be dependent on peripheral immunity. Consistent with the idea that microglia do not regulate GFAP + astrocyte numbers or astrocyte recruitment to amyloid plaques, Kianai Shabestari et al. (2022) found that microglial-specific depletion in 5XFAD mice crossed with CSF1R FIRE enhancer knockout mice, did not influence GFAP + astrocyte numbers or PAAs [94]. In contrast to these phenotypes, our data suggest that GMB-mediated alteration in astrocyte morphology are at least partially dependent on microglia. The microglial dependent GMB-mediated astrocyte morphology phenotype contributes to growing literature suggesting that microglia-astrocyte crosstalk is important in neuroinflammatory response to pathology [11, 55–58, 95, 96]. Herein, we have tested how microglial absence alters GMB-mediated regulation of astrocyte response in the context of amyloidosis. It will also be interesting to test how alteration of astrocyte activity or depletion of reactive astrocyte subtypes impacts microglial responses to GMB perturbation. Overall, our results suggest the presence of both microglial independent and microglial dependent GMB regulation of reactive astrocytosis. Future studies will delineate which specific astrocytic molecular pathways governed by the GMB are microglial independent and dependent.

While the blood-based link between the GMB and astrocytes has not yet been elucidated, it is likely that GMB-derived metabolites may have an effect on peripheral immunity and potentially CNS immunity as well. Spichak and colleagues found that short chain fatty acids can regulate astrocyte gene expression in a sex-specific manner [80]. This study showed acetate induces GFAP expression in male but not female primary astrocytes. Rothhammer and colleagues found that gut-derived metabolites of dietary tryptophan limit astrocyte inflammatory activity directly through aryl hydrocarbon receptor mediated IFN-I signaling in the experimental autoimmune encephalitis (EAE) model of Multiple Sclerosis [97]. It is possible that differential metabolism of tryptophan after abx treatment may be the mechanism reducing GFAP + astrocyte inflammatory activity and amyloidosis in our model. However, EAE induces acute substantial peripheral and central inflammation, which is quite different than the more chronic amyloidosis model-associated inflammation which takes place over months [98]. Future studies will need to be done to study the role of tryptophan-derived metabolites in the connection between GMB perturbation and astrocyte phenotype.

Although abx is protective in the APPPS1-21 model in terms of amyloidosis, it is likely more useful as a model of GMB perturbation rather than as a therapeutic strategy for AD. It is possible that early life antibiotic use is associated with a decreased risk of developing AD. However, several adverse pathologies are associated with early life antibiotics use, such as asthma, celiac disease, and obesity [99]. Similarly, it is possible that late life antibiotic treatment could alter amyloid load, but this has not been extensively tested. We would expect that long term abx use in elderly mice or humans may alter amyloid load and neuroinflammation, but there are few instances in which humans take long-term abx. However, we suspect that long-term abx use will likely increase risk for other pathologies, so it would not be a viable therapeutic strategy [99]. In addition to abx, several studies have reported that probiotics are beneficial in AD mouse models and in small trials in human AD patients [100–102]. Furthermore, studies in mouse models have also shown that FMT treatment from a healthy donor could be beneficial in human AD [103]. GMB-targeted therapy will likely come in the form of probiotic/prebiotic or FMT treatment since primarily beneficial strains of bacteria would be amplified in this strategy. Additionally, further understanding of the mechanisms whereby the GMB alters AD progression may lead to therapeutic targeting of GMB regulated neuroinflammatory pathways.

Our study has some limitations, which center around evaluation of astrocyte phenotypes. Herein, we have evaluated astrocyte morphology and GFAP/C3 reactivity phenotypes. Although previous studies have associated morphologies and GFAP/C3 positivity with reactive astrocytes, these endpoints do not fully elucidate how the GMB alters astrocyte functionality. We took initial steps to answer this question by utilizing previously published astrocyte-relevant gene modules [52, 53] to assess bulk RNA sequencing data generated in Dodiya et al. 2022 [27] (abx vs vhl). We found a decrease of gene modules associated with inflammation (amyloid, LPS) and an increase in gene modules associated with neuroprotection (MCAO) in abx treated mice. However, several astrocyte-relevant genes are also expressed by other cell types. Future studies will require astrocyte-specific transcriptomic, spatial, and functional experiments to fully elucidate how the GMB alters astrocytes phenotype in this model. We may find that abx-treated astrocytes are anti-inflammatory or have a difference in lysosomal function similar to other studies [22]. Additionally, although we have shown that microglia are likely important in facilitating GMB perturbation influence on some astrocyte phenotypes, it is possible that other cell types are important in controlling astrocyte phenotype, such as natural killer cells [22] or other peripheral immune cells, but this needs to be investigated in future studies.

Another limitation of our study is that CSF1R inhibitor PLX5622 does not selectively deplete brain resident microglia but can also deplete peripheral myeloid lineage cells [104]. We cannot rule out the possibility that peripheral myeloid cells may be contributing to the phenotypes observed in our PLX experiments. Future studies utilizing CSF1R FIRE enhancer knockout mice [94, 105], which lack microglia but retain peripheral myeloid cells, may better delineate if the PLX-mediated phenotypes observed in this study are due to depletion of microglia or if they are actually due to depletion of peripheral macrophages and monocytes.

In future studies, it will be important to understand the mechanism by which GMB-mediated astrocytosis may contribute to amyloidosis. It is possible that the GMB may regulate amyloid processing genes, such as BACE1 or APP, in astrocytes which may directly impact amyloid generation. However, previous studies have shown that whole brain levels of BACE1 and APP are not changed after abx treatment [25]. However, it is unknown whether expression of these genes is altered by abx treatment in astrocytes specifically. Our data suggests that the GMB regulates inflammatory pathways in astrocytes which contribute to amyloidosis, such as the JAK/STAT pathway [106] or NFAT pathway [107]. For example, it is possible that abx diminishes JAK/STAT signaling in astrocytes, which has been shown to potentiate amyloid plaque pathology [84, 106]. Additionally, reactive astrocytes can upregulate amyloid processing machinery [108] or release cholesterol or interferon induced transmembrane protein 3 to alter amyloid processing machinery in neurons [109, 110]. Future transcriptomic study of astrocytes after abx treatment and subsequent functional experiments will be important for determining the exact mechanism whereby GMB control of astrocyte phenotype impacts amyloidosis.

Our studies show that abx and GF conditions diminish astrocytic association with amyloid plaques, and FMT treatment after abx treatment restores astrocyte plaque association. These results suggest that the GMB is an upstream mediator of astrocytic recruitment to amyloid plaques. There are several receptors that may mediate astrocytic recognition of amyloid beta, such as, *Ldlr *[111, 112]*, Lrp4 *[113]*, Lrp1 *[114]*, and Megf10 *[115]*,* that may be downregulated after abx-treatment resulting in the inability of the astrocyte to be recruited to the amyloid plaque. This will be an important topic for future study.

Another limitation of our study is that the time points evaluated are 9 weeks and 3 months when the APPPS1-21 mouse is still in the growth phase of plaque accumulation. We selected these time points to assess how early astrocytic responses to amyloid plaques are impacted by GMB perturbations. However, in future studies it will be of interest to see if this response persists into late age after early life abx treatment. Since the effect of early life abx persists from 9 weeks to 3 months and other studies have shown persistent effects on amyloidosis and microgliosis [24, 34, 41] with age after GMB alteration, we would suspect that we will observe the same phenomenon in astrocytes with increased aging.

An interesting question that this study brings up is whether administration of abx in amyloid model mice once the plaques have formed would still be protective. In the current study and most other studies testing abx in amyloid models, abx has been given to mice when they are young and still have a relatively dynamic GMB [24–27, 77]. In contrast, adult humans and adult mice have a more stable GMB [116]. To our knowledge, there is only one current published study that has evaluated whether abx treatment after the start of amyloidosis has the same effect as it has before amyloidosis [41]. Mezo et al. [41] found that 2 months of abx treatment at 2 months of age in the 5XFAD model when plaques start to form and treatment at 8 months when there is a heavy plaque load, still resulted in a reduction of plaques when assessed at 4 months and 10 months respectively. This result suggests that GMB changes in both early and late life are relevant to modifying AD risk. However, it may be that more significant GMB changes are necessary to modify AD risk and progression in late life than early life.

Overall, we report that GMB perturbation via a short-term abx cocktail and GMB absence via GF environment causes significant reductions in GFAP + reactive astrocytosis, astrocyte recruitment to plaques, astrocytic C3 levels, and increases homeostatic astrocytic morphologic complexity in APPPS1-21 male mice. FMT from donor APPPS1-21 male mice into abx-treated APPPS1-21 male mice reversed astrocyte phenotypes confirming that abx effects on astrocytes are GMB specific. We found correlations between particular bacterial genera that are depleted by abx treatment and astrocyte phenotypes, suggesting that these pathogenic bacteria may regulate astrocyte phenotypes and contribute to Aβ pathology. Lastly, studies using CSF1R inhibition in vehicle and abx treated APPPS1-21 male mice showed that GMB regulation of reactive astrocytosis is governed by both microglial independent and dependent mechanisms. Overall, our results indicate GMB modulates reactive astrocyte induction and reaction to Aβ plaque pathology. Future studies elucidating which pathways in astrocytes are modulated by the GMB could result in drug candidates for astrocyte-specific therapeutic intervention for AD.

## Conclusions

Our study demonstrates that the GMB has a role in modulating reactive astrocyte induction in the context of amyloidosis. Reactive astrocyte induction, recruitment to Aβ plaques, and astrocytic C3 levels are reduced and homeostatic astrocytic morphologic complexity is increased upon abx-mediated GMB depletion or in the absence of the GMB in germ-free environments. Importantly, restoration of the GMB with FMT from untreated donor male mice into abx-treated mice reverses astrocyte phenotypes. Using CSF1R inhibition in vehicle or abx treated mice, we demonstrate that the GMB regulates astrocyte phenotypes through both microglial independent and dependent mechanisms. Our study provides the precedence for future study of GMB-controlled reactive astrocytosis in AD.

## Supplementary Information

**Additional file 1: Supplementary Figure 1.** short-term abx increases cecum weight in APPPS1-21 male and female mice.Comparison of body weight,cecum weight, andcecum/body weight ratio between VHL treated male APPPS1-21 mice and ABX treated male APPPS1-21 mice.Comparison of body weight,cecum weight, andcecum/body weight ratio between VHL treated female APPPS1-21 mice and ABX treated female APPPS1-21 mice. M=male. Data expressed as mean +/- standard deviation; VHL M N= 7, ABX M N= 8, VHL F N= 8, ABX F N= 8. Statistics calculated using two-tailed unpaired student’s t-tests. * denotes a *p*-value ≤0.05, ** indicates *p*-value ≤0.01, *** indicates *p*-value ≤0.001, and **** indicates a *p*-value of ≤ 0.0001.

**Additional file 2: Supplemental Figure 2.** Administration of short-term antibiotics alters gut microbiota profile in male and female APPPS1-21 mice.PCA plot of 16s rRNAseq profiling from male and female VHL and ABX treated mouse fecal samples comparing PC1 vs PC2,PC1 vs PC3, andPC2 vs PC3.Alpha diversityanalysis comparing gut microbiota in male VHL and ABX treated mice.Alpha diversityanalysis comparing gut microbiota in female VHL and ABX treated mice.Beta diversity analysis comparing gut microbiota in male and female VHL and ABX treated mice. VHL M N= 6, ABX M N= 7, VHL F N= 8, ABX F N= 8. Alpha diversity pairwise statistics calculated using Mann-Whitney test. VHL vs ABX Male *p*-value=0.0082 and VHL vs ABX female *p*-value=0.65. Beta diversity pairwise statistics calculated using ANOSIM. VHL vs ABX Male and female *p*-value=0.002.

**Additional file 3: Supplemental Figure 3.** short-term abx alters several genera in APPPS1-21 mice. Quantification of 16s rRNAseq sequence abundance ofOdoribacter,Muribaculum,Akkermansia,Pararevotella,Prevotellaceae,Millionella,Lachnospiraceae,Tyzzerella,Intestinimonas, andAnaeroplasma in ABX female, VHL female, ABX male, and VHL male groups. FDR p-adj value of ≤0.05 was used to identify statistical significance. VHL M N= 6, ABX M N= 7, VHL F N= 8, ABX F N= 8.

**Additional file 4: Supplemental Figure 4.** gut microbiome perturbations alter Aβ pathology in APPPS1-21 mice.Quantification of amyloid plaques/µm2,amyloid plaque percent area,and average plaque sizein 9-week-old VHL male and ABX male APPPS1-21 mice.Quantification of amyloid plaques/µm2,amyloid plaque percent area, andaverage plaque sizein VHL female and ABX female APPPS1-21 mice.Quantification of amyloid plaques/µm2,amyloid plaque percent area, andaverage plaque sizein ABX male and ABX+ FMT male APPPS1-21 mice.Quantification of amyloid plaques/µm2,amyloid plaque percent area, andaverage plaque sizein SPF male and GF male APPPS1-21 mice.Quantification of amyloid plaques/µm2,amyloid plaque percent area,and average plaque sizein VHL male and ABX 3 month old male APPPS1-21 mice.Quantification of amyloid plaques/µm2,amyloid plaque percent area,and average plaque sizein PLX male and PLX+ ABX 3-month-old male APPPS1-21 mice. M=male, F=female. Data expressed as mean +/- standard deviation; N=7-10/group. Statistics calculated using two-tailed unpaired student’s t-tests. 4 sections used per animal. * denotes a *p*-value ≤0.05, ** indicates *p* value ≤0.01, *** indicates *p*-value ≤0.001, and **** indicates a *p*-value of ≤ 0.0001.

**Additional file 5: Supplemental Figure 5.** Cropped immunoblots quantified in Figure 1.GFAP, IBA1, and β-actin immunoblot comparing VHL M and ABX M which is quantified in Figure 1.GFAP and β actin immunoblot comparing VHL F and ABX F which is quantified in Figure 1.IBA1 and β-actin immunoblot comparing VHL F and ABX F which is quantified in Figure 1.

**Additional file 6: Supplemental Figure 6.** Uncropped immunoblots quantified in Figure 1.Uncropped immunoblot for GFAP,IBA1, andβ-actin protein levels compared between VHL and ABX treated male mice. Uncropped immunoblot forGFAP,β-actin,IBA1, andβ-actinprotein levels compared between VHL and ABX treated female mice. Cropped  blots for A-G appear in Supplemental Figure 5 and quantifications are in Figure 1. Uncropped immunoblot forEzrin,IL33, andβ-actin protein levels compared between VHL and ABX treated male mice. Cropped blots and quantifications for H-J appear in Figure 2.

**Additional file 7: Supplemental Figure 7.** S100B+ Sox9+ astrocytes/µm2 levels are not altered by abx treatment in male APPPS1-21 mice.Representative Sox9+, S100B+, Sox9+ S100B+, DAPI+, and Sox9+ S100B+ DAPI+cell images from VHL maleand ABX male.Quantification of Sox9+ DAPI+ cells/µm2,S100B+ DAPI+ cells/µm2,Sox9+ S100B+ DAPI+ cells/µm2, andmean intensity of S100B in Sox9+ S100B+ DAPI+ cells. M=male. Data expressed as mean +/- standard deviation. VHL M N= 7, ABX M N= 7. Statistics calculated using two-tailed unpaired student’s t-tests. 4 sections used per animal. Scale bars indicate 50 µm.

**Additional file 8: Supplemental Figure 8.** Administration of short-term antibiotics does not alter astrocyte morphology or C3+ astrocyte reactivity in the brain of female APPPS1-21 mice.Representative GFAP, C3, and Aβ merged astrocyte z-stack maximum projections, IMARIS 3D reconstructions, and IMARIS filament 3D reconstructionsfor VHL femaleand ABX femalegroups. GFAP, C3, and Aβshown as separate channels from merged images.Quantification and comparison of astrocyte mean process length sum,astrocyte mean process area sum,astrocyte mean process volume sum, astrocyte soma area/GFAP area,astrocyte C3 area/GFAP area,astrocyte mean number of processes,astrocyte mean number of process branch points, andastrocyte mean number of process terminal points between VHL female and ABX female groups. F=female. Data expressed as mean +/- standard deviation. VHL F N= 8, ABX F N= 8. Statistics calculated using two-tailed unpaired student’s t-tests. 4 sections used per animal. * denotes a *p*-value ≤0.05, ** indicates *p* value ≤0.01, *** indicates *p*-value ≤0.001, and **** indicates a *p*-value of ≤ 0.0001. Scale bars indicate 20 µm.

**Additional file 9: Supplemental Figure 9.** Abx-mediated alterations in astrocyte-associated transcripts. Volcano plot of previously identified amyloid-induced, LPS induced, MCAO induced, pan-reactive, aging-induced, and aging-reducedastrocyte genes in male APPPS1-21 abx vs vehicle-treated RNAseq data from Dodiya et al 2022 [27]. Quantitative polymerase chain reactionexpression of H2-d1Emp1and Cxcl10genes in male APPPS1-21 mice treated with abx or water vehicle control. Amyloid induced astrocyte genes were identified in Jiwaji et al 2022 [52]. LPS-induced, MCAO-induced, and pan-reactive astrocyte gene sets were originally identified in Zamanian et al 2012 [53] but were reanalyzed by Jiwaji et al 2022 [52] to generate larger gene lists with more clear rationales of how genes were sorted in each category from the original data. Aging induced and reduced astrocyte genes were identified in Clarke et al 2018 [54]. Dotted lines in volcano plots correspond with a FDR p-adj value of ≤0.05.

**Additional file 10.** Antibodies used in study. This table contains the primary and secondary antibodies used for this study, vendors, and concentrations at which the antibodies were used.

**Additional file 11.** Gut microbiome 16s rRNA amplicon differential expression analysis. This table contains the results of differential expression analysis of 16s rRNA amplicon sequencing from male and female animals treated with antibiotics or vehicle.

**Additional file 12.** Pearson’s correlation coefficients and p-values for correlation matrix analysis between abx-altered bacterial genera and astrocyte phenotypes. This table contains Pearson’s correlation coefficients and p-values for correlation matrix analysis between abx-altered bacterial genera and astrocyte phenotypes for male APPPS1-21 mice.

## Data Availability

Bulk RNAseq data referenced in Supplemental Fig. 9 can be accessed at Gene Omnibus Number GSE185407 (Dodiya et al. 2022) and European Nucleotide Archive E-MTAB-10985 (Jiwaji et al. 2022). All datasets generated are included in this article and the supplemental information files.

## Abbreviations

Aβ: Amyloid beta
AD: Alzheimer’s disease
ABX: Antibiotics
CSF1R: Colony-stimulating factor 1 receptor
DAM: Disease-associated microglia
FMT: Fecal matter transplant
GF: Germ-free
GMB: Gut microbiome
GFAP: Glial fibrillary acidic protein
IHC: Immunohistochemistry
rRNA: Ribosomal RNA
PAAs: Plaque-associated astrocytes
SPF: Specific pathogen free
