# Supplementary figures and images for: The gut microbiome regulates astrocyte reaction to Aβ amyloidosis through microglial dependent and independent mechanisms
Source: Mol Neurodegener. 2023 Jul 6;18:45. doi: 10.1186/s13024-023-00635-2 (PMC10324210; doi:10.1186/s13024-023-00635-2)

Male

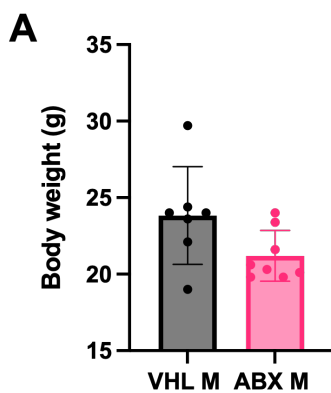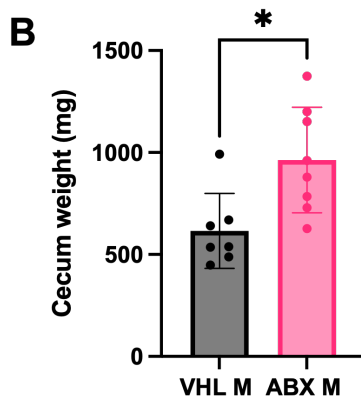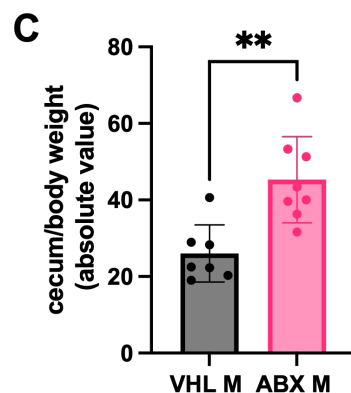

Female

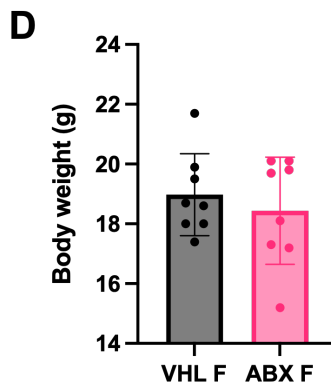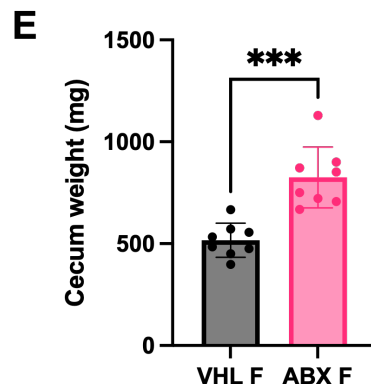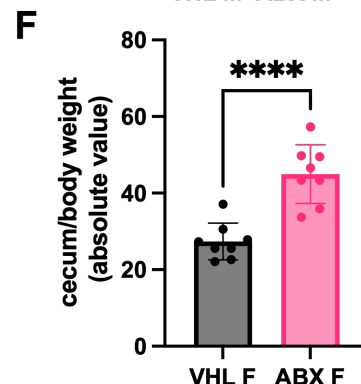

Supplement: Supplementary file 1 — Additional file 1: Supplementary Figure 1. short-term abx increases cecum weight in APPPS1-21 male and female mice.Comparison of body weight,cecum weight, andcecum/body weight ratio between VHL treated male APPPS1-21 mice and ABX treated male APPPS1-21 mice.Comparison of body weight,cecum weight, andcecum/body weight ratio between VHL treated female APPPS1-21 mice and ABX treated female APPPS1-21 mice. M=male. Data expressed as mean +/- standard deviation; VHL M N= 7, ABX M N= 8, VHL F N= 8, ABX F N= 8. Statistics calculated using two-tailed unpaired student’s t-tests. * denotes a p-value ≤0.05, ** indicates p-value ≤0.01, *** indicates p-value ≤0.001, and **** indicates a p-value of ≤ 0.0001. [file 13024_2023_635_MOESM1_ESM.pdf]

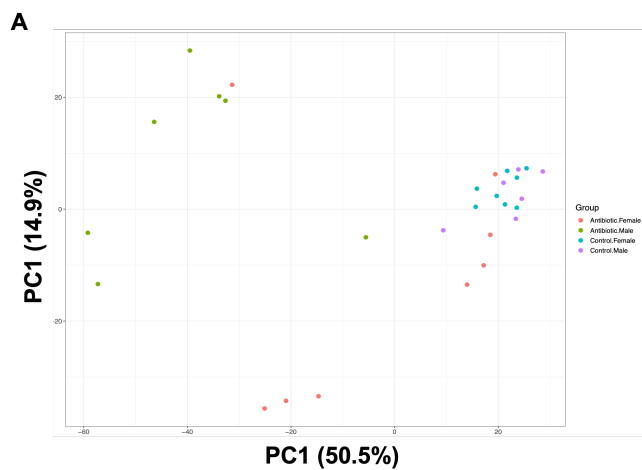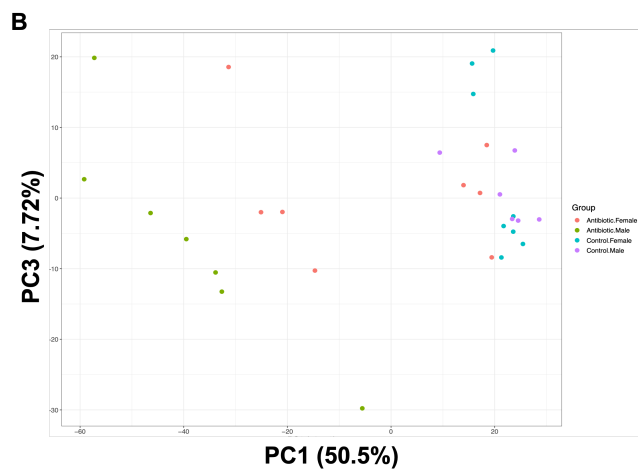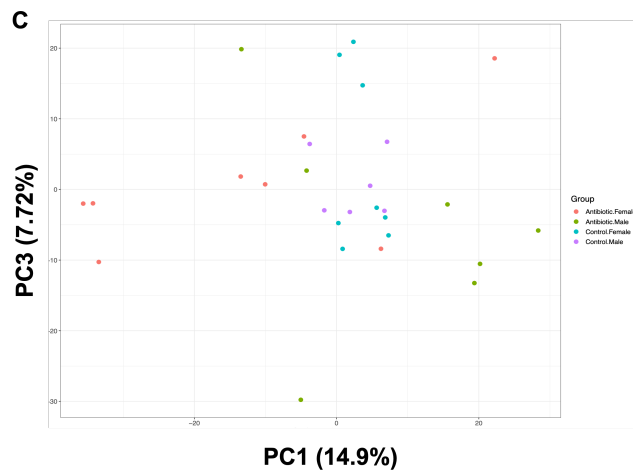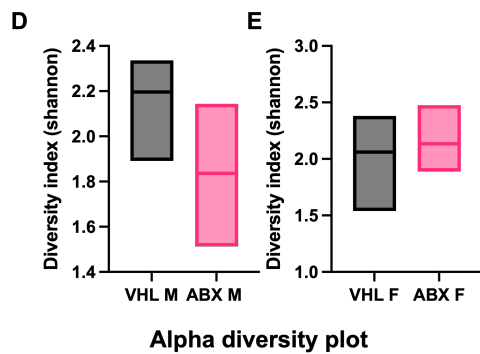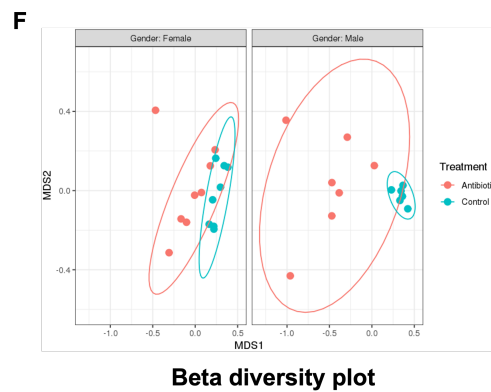

Supplement: Supplementary file 2 — Additional file 2: Supplemental Figure 2. Administration of short-term antibiotics alters gut microbiota profile in male and female APPPS1-21 mice.PCA plot of 16s rRNAseq profiling from male and female VHL and ABX treated mouse fecal samples comparing PC1 vs PC2,PC1 vs PC3, andPC2 vs PC3.Alpha diversityanalysis comparing gut microbiota in male VHL and ABX treated mice.Alpha diversityanalysis comparing gut microbiota in female VHL and ABX treated mice.Beta diversity analysis comparing gut microbiota in male and female VHL and ABX treated mice. VHL M N= 6, ABX M N= 7, VHL F N= 8, ABX F N= 8. Alpha diversity pairwise statistics calculated using Mann-Whitney test. VHL vs ABX Male p-value=0.0082 and VHL vs ABX female p-value=0.65. Beta diversity pairwise statistics calculated using ANOSIM. VHL vs ABX Male and female p-value=0.002. [file 13024_2023_635_MOESM2_ESM.pdf]

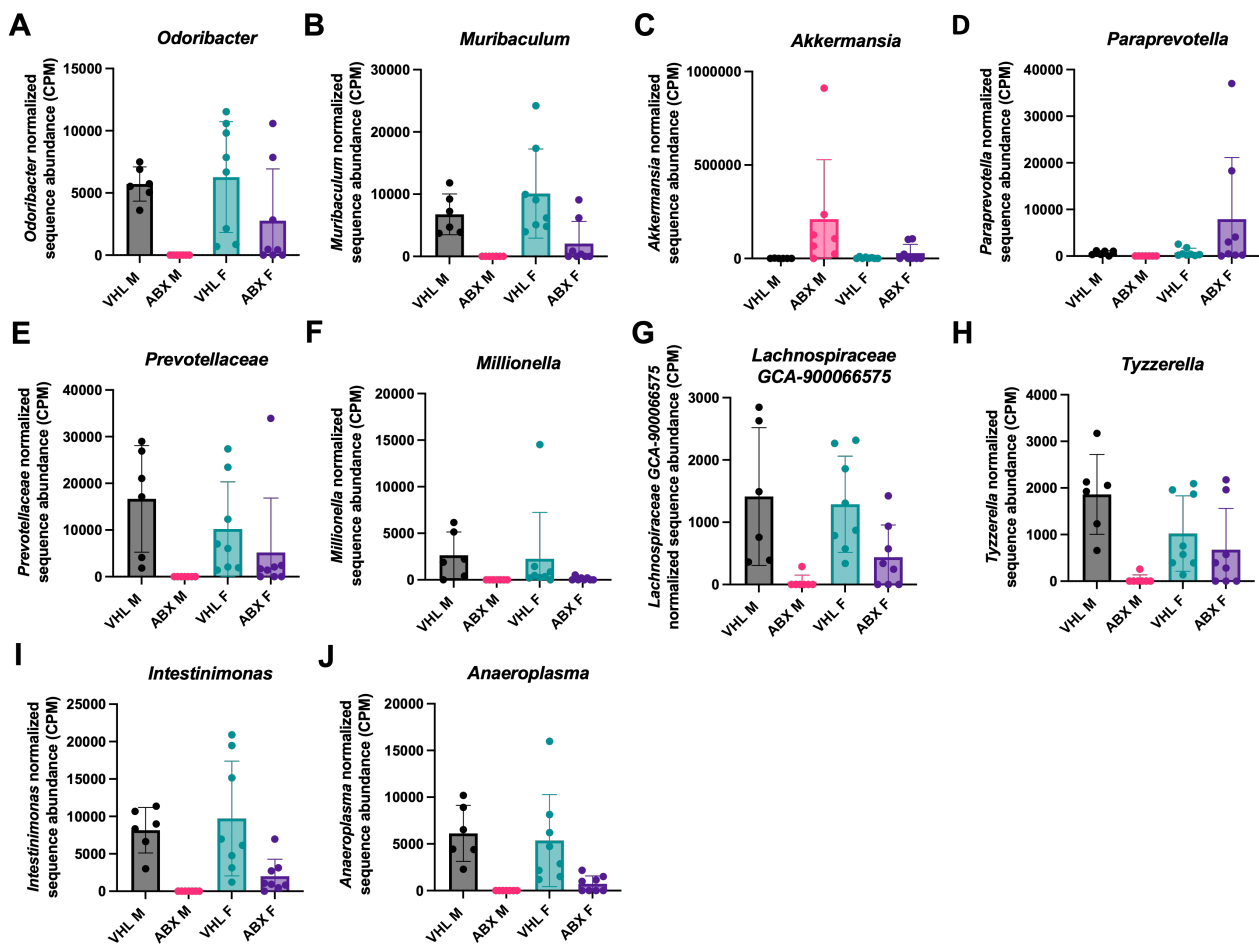

Supplement: Supplementary file 3 — Additional file 3: Supplemental Figure 3. short-term abx alters several genera in APPPS1-21 mice. Quantification of 16s rRNAseq sequence abundance ofOdoribacter,Muribaculum,Akkermansia,Pararevotella,Prevotellaceae,Millionella,Lachnospiraceae,Tyzzerella,Intestinimonas, andAnaeroplasma in ABX female, VHL female, ABX male, and VHL male groups. FDR p-adj value of ≤0.05 was used to identify statistical significance. VHL M N= 6, ABX M N= 7, VHL F N= 8, ABX F N= 8. [file 13024_2023_635_MOESM3_ESM.pdf]

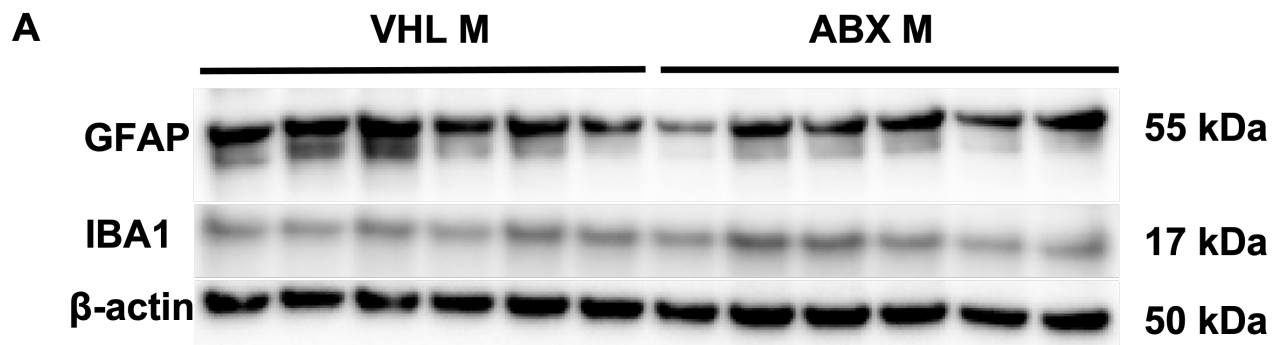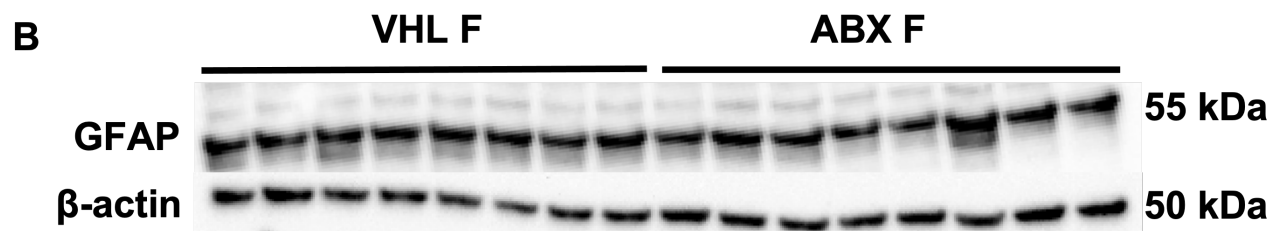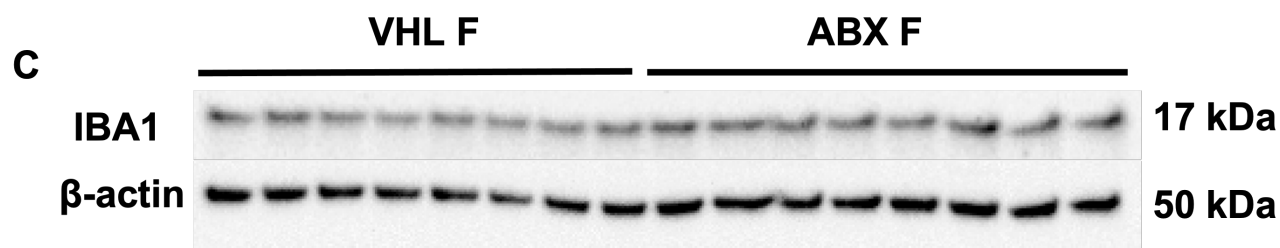

Supplement: Supplementary file 5 — Additional file 5: Supplemental Figure 5. Cropped immunoblots quantified in Figure 1.GFAP, IBA1, and β-actin immunoblot comparing VHL M and ABX M which is quantified in Figure 1.GFAP and β actin immunoblot comparing VHL F and ABX F which is quantified in Figure 1.IBA1 and β-actin immunoblot comparing VHL F and ABX F which is quantified in Figure 1. [file 13024_2023_635_MOESM5_ESM.pdf]

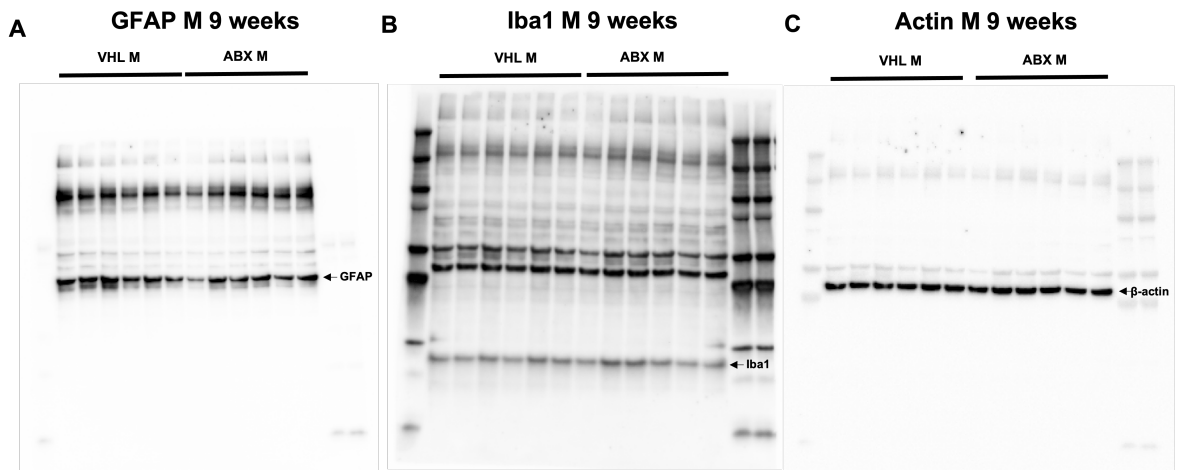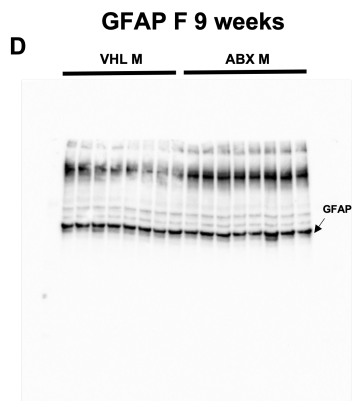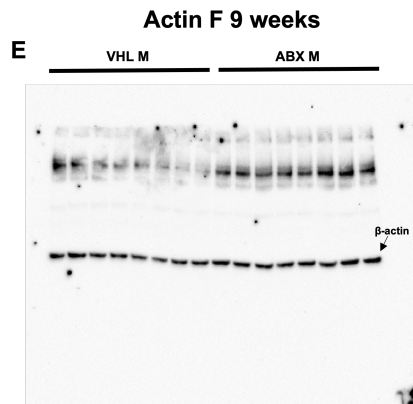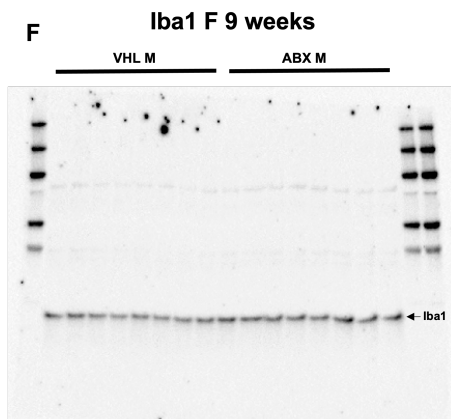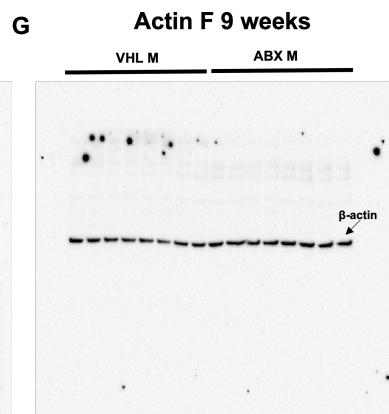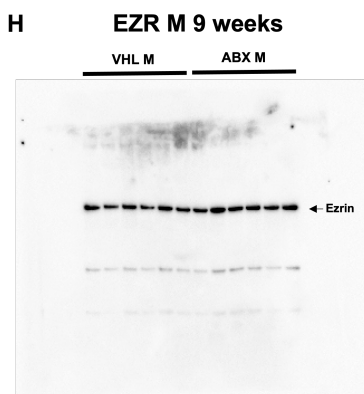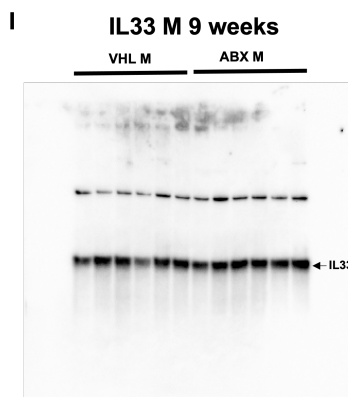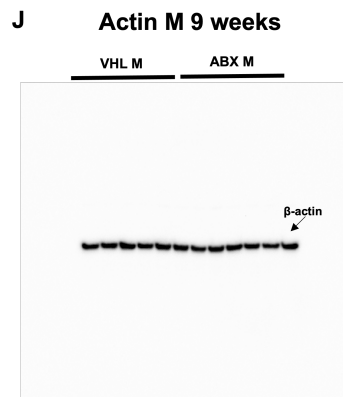

Supplement: Supplementary file 6 — Additional file 6: Supplemental Figure 6. Uncropped immunoblots quantified in Figure 1.Uncropped immunoblot for GFAP,IBA1, andβ-actin protein levels compared between VHL and ABX treated male mice. Uncropped immunoblot forGFAP,β-actin,IBA1, andβ-actinprotein levels compared between VHL and ABX treated female mice. Cropped blots for A-G appear in Supplemental Figure 5 and quantifications are in Figure 1. Uncropped immunoblot forEzrin,IL33, andβ-actin protein levels compared between VHL and ABX treated male mice. Cropped blots and quantifications for H-J appear in Figure 2. [file 13024_2023_635_MOESM6_ESM.pdf]

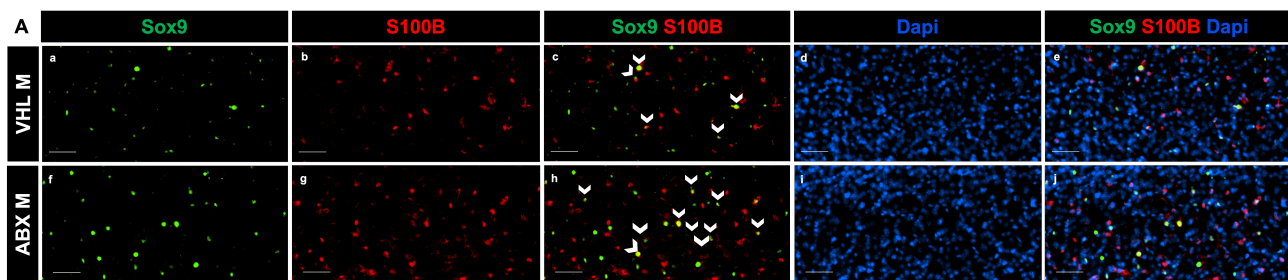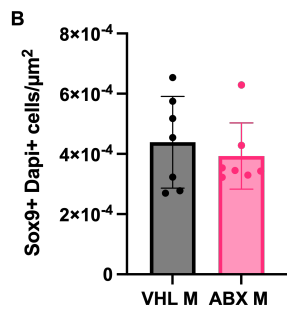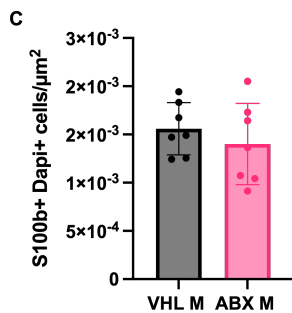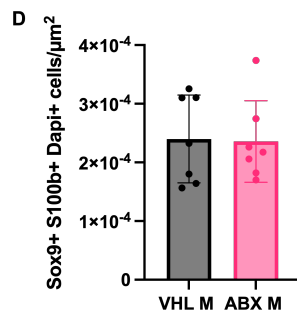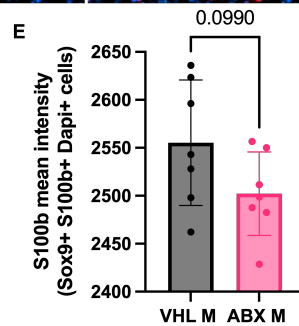

Supplement: Supplementary file 7 — Additional file 7: Supplemental Figure 7. S100B+ Sox9+ astrocytes/µm2 levels are not altered by abx treatment in male APPPS1-21 mice.Representative Sox9+, S100B+, Sox9+ S100B+, DAPI+, and Sox9+ S100B+ DAPI+cell images from VHL maleand ABX male.Quantification of Sox9+ DAPI+ cells/µm2,S100B+ DAPI+ cells/µm2,Sox9+ S100B+ DAPI+ cells/µm2, andmean intensity of S100B in Sox9+ S100B+ DAPI+ cells. M=male. Data expressed as mean +/- standard deviation. VHL M N= 7, ABX M N= 7. Statistics calculated using two-tailed unpaired student’s t-tests. 4 sections used per animal. Scale bars indicate 50 µm. [file 13024_2023_635_MOESM7_ESM.pdf]

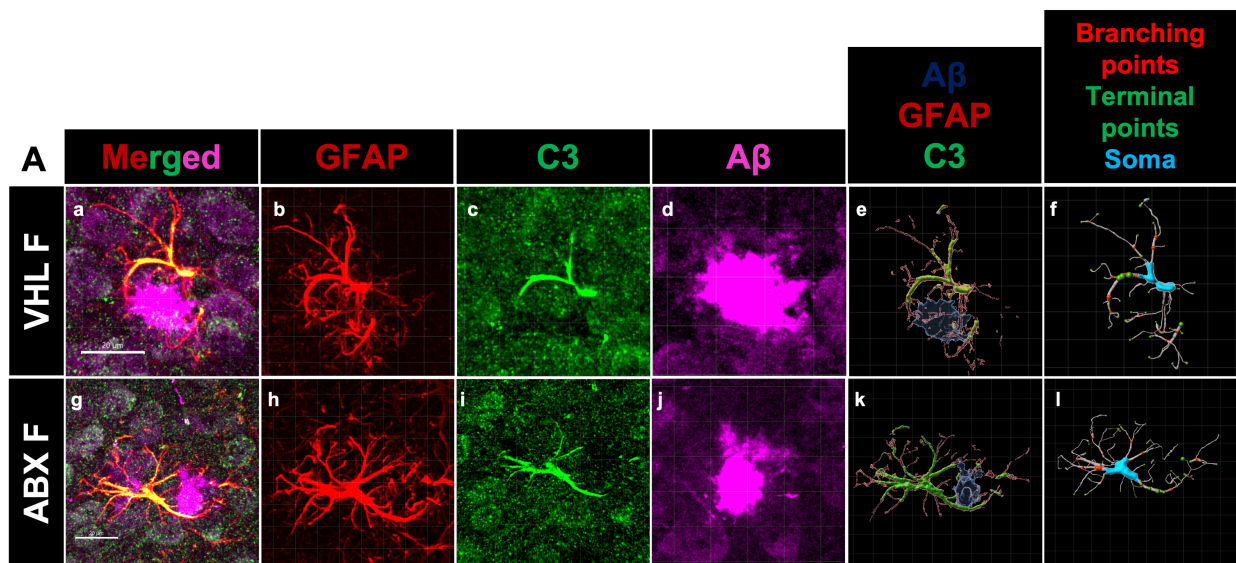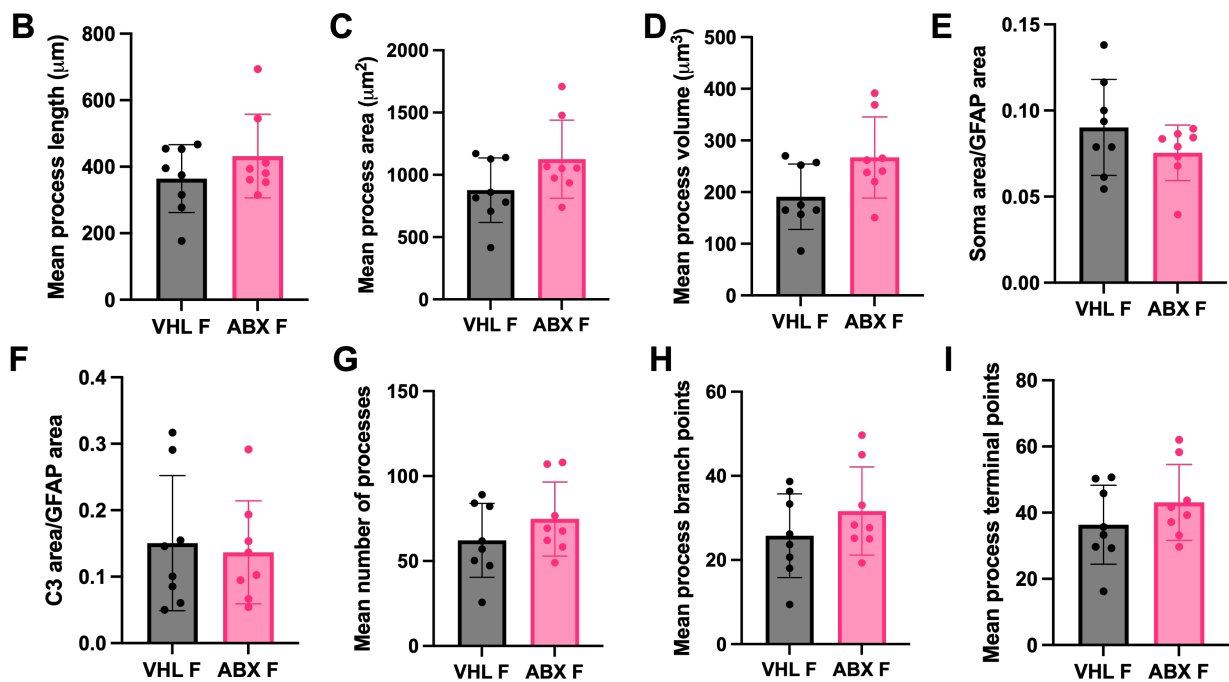

Supplement: Supplementary file 8 — Additional file 8: Supplemental Figure 8. Administration of short-term antibiotics does not alter astrocyte morphology or C3+ astrocyte reactivity in the brain of female APPPS1-21 mice.Representative GFAP, C3, and Aβ merged astrocyte z-stack maximum projections, IMARIS 3D reconstructions, and IMARIS filament 3D reconstructionsfor VHL femaleand ABX femalegroups. GFAP, C3, and Aβshown as separate channels from merged images.Quantification and comparison of astrocyte mean process length sum,astrocyte mean process area sum,astrocyte mean process volume sum, astrocyte soma area/GFAP area,astrocyte C3 area/GFAP area,astrocyte mean number of processes,astrocyte mean number of process branch points, andastrocyte mean number of process terminal points between VHL female and ABX female groups. F=female. Data expressed as mean +/- standard deviation. VHL F N= 8, ABX F N= 8. Statistics calculated using two-tailed unpaired student’s t-tests. 4 sections used per animal. * denotes a p-value ≤0.05, ** indicates p value ≤0.01, *** indicates p-value ≤0.001, and **** indicates a p-value of ≤ 0.0001. Scale bars indicate 20 µm. [file 13024_2023_635_MOESM8_ESM.pdf]

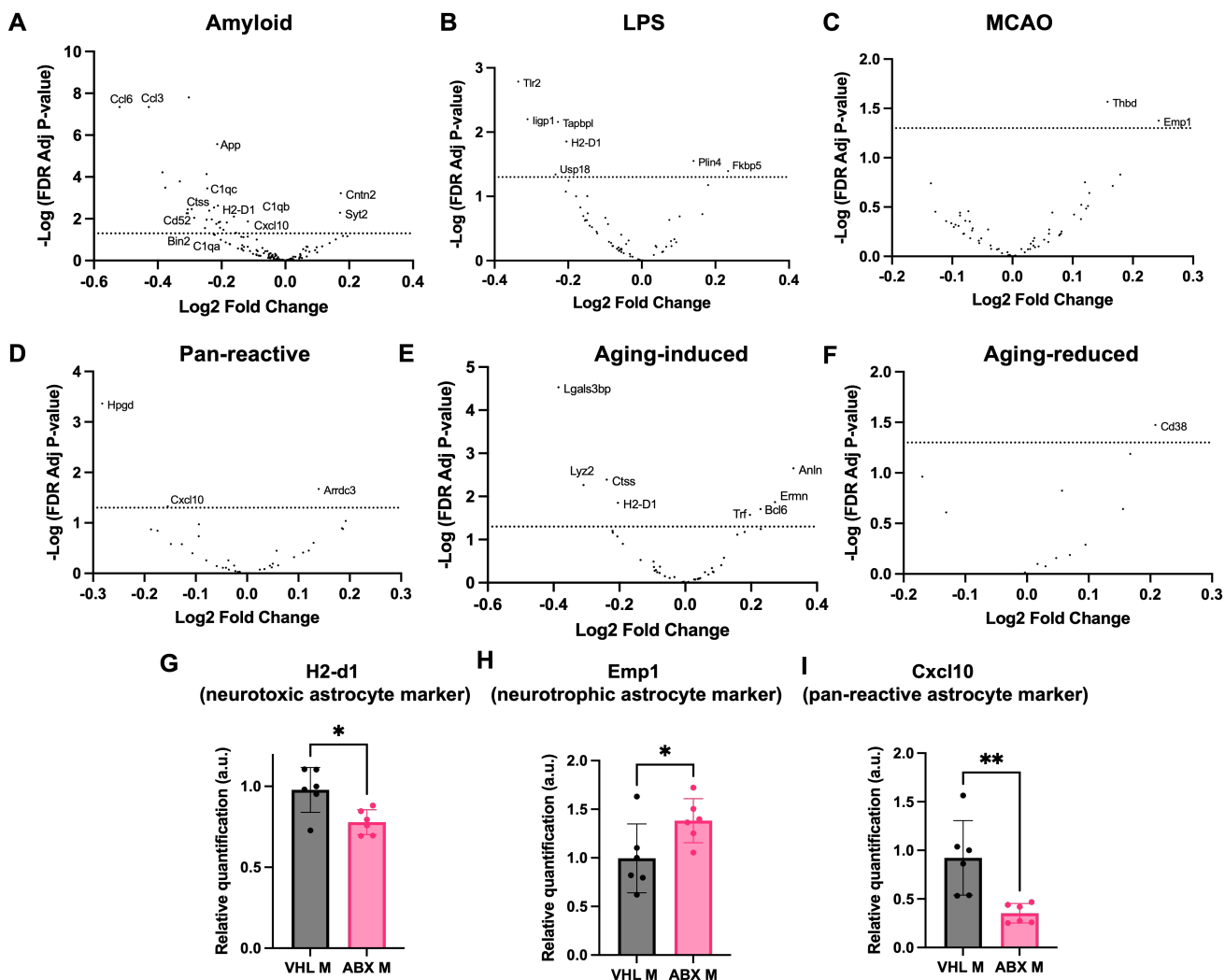

Supplement: Supplementary file 9 — Additional file 9: Supplemental Figure 9. Abx-mediated alterations in astrocyte-associated transcripts. Volcano plot of previously identified amyloid-induced, LPS induced, MCAO induced, pan-reactive, aging-induced, and aging-reducedastrocyte genes in male APPPS1-21 abx vs vehicle-treated RNAseq data from Dodiya et al 2022 [27]. Quantitative polymerase chain reactionexpression of H2-d1Emp1and Cxcl10genes in male APPPS1-21 mice treated with abx or water vehicle control. Amyloid induced astrocyte genes were identified in Jiwaji et al 2022 [52]. LPS-induced, MCAO-induced, and pan-reactive astrocyte gene sets were originally identified in Zamanian et al 2012 [53] but were reanalyzed by Jiwaji et al 2022 [52] to generate larger gene lists with more clear rationales of how genes were sorted in each category from the original data. Aging induced and reduced astrocyte genes were identified in Clarke et al 2018 [54]. Dotted lines in volcano plots correspond with a FDR p-adj value of ≤0.05. [file 13024_2023_635_MOESM9_ESM.pdf]
