# Supplementary material for: The gut microbiome regulates astrocyte reaction to Aβ amyloidosis through microglial dependent and independent mechanisms
Source: Mol Neurodegener. 2023 Jul 6;18:45. doi: 10.1186/s13024-023-00635-2 (PMC10324210; doi:10.1186/s13024-023-00635-2)
Supplement: Supplementary file 4 — Additional file 4: Supplemental Figure 4. gut microbiome perturbations alter Aβ pathology in APPPS1-21 mice.Quantification of amyloid plaques/µm2,amyloid plaque percent area,and average plaque sizein 9-week-old VHL male and ABX male APPPS1-21 mice.Quantification of amyloid plaques/µm2,amyloid plaque percent area, andaverage plaque sizein VHL female and ABX female APPPS1-21 mice.Quantification of amyloid plaques/µm2,amyloid plaque percent area, andaverage plaque sizein ABX male and ABX+ FMT male APPPS1-21 mice.Quantification of amyloid plaques/µm2,amyloid plaque percent area, andaverage plaque sizein SPF male and GF male APPPS1-21 mice.Quantification of amyloid plaques/µm2,amyloid plaque percent area,and average plaque sizein VHL male and ABX 3 month old male APPPS1-21 mice.Quantification of amyloid plaques/µm2,amyloid plaque percent area,and average plaque sizein PLX male and PLX+ ABX 3-month-old male APPPS1-21 mice. M=male, F=female. Data expressed as mean +/- standard deviation; N=7-10/group. Statistics calculated using two-tailed unpaired student’s t-tests. 4 sections used per animal. * denotes a p-value ≤0.05, ** indicates p value ≤0.01, *** indicates p-value ≤0.001, and **** indicates a p-value of ≤ 0.0001. [file 13024_2023_635_MOESM4_ESM.pdf]

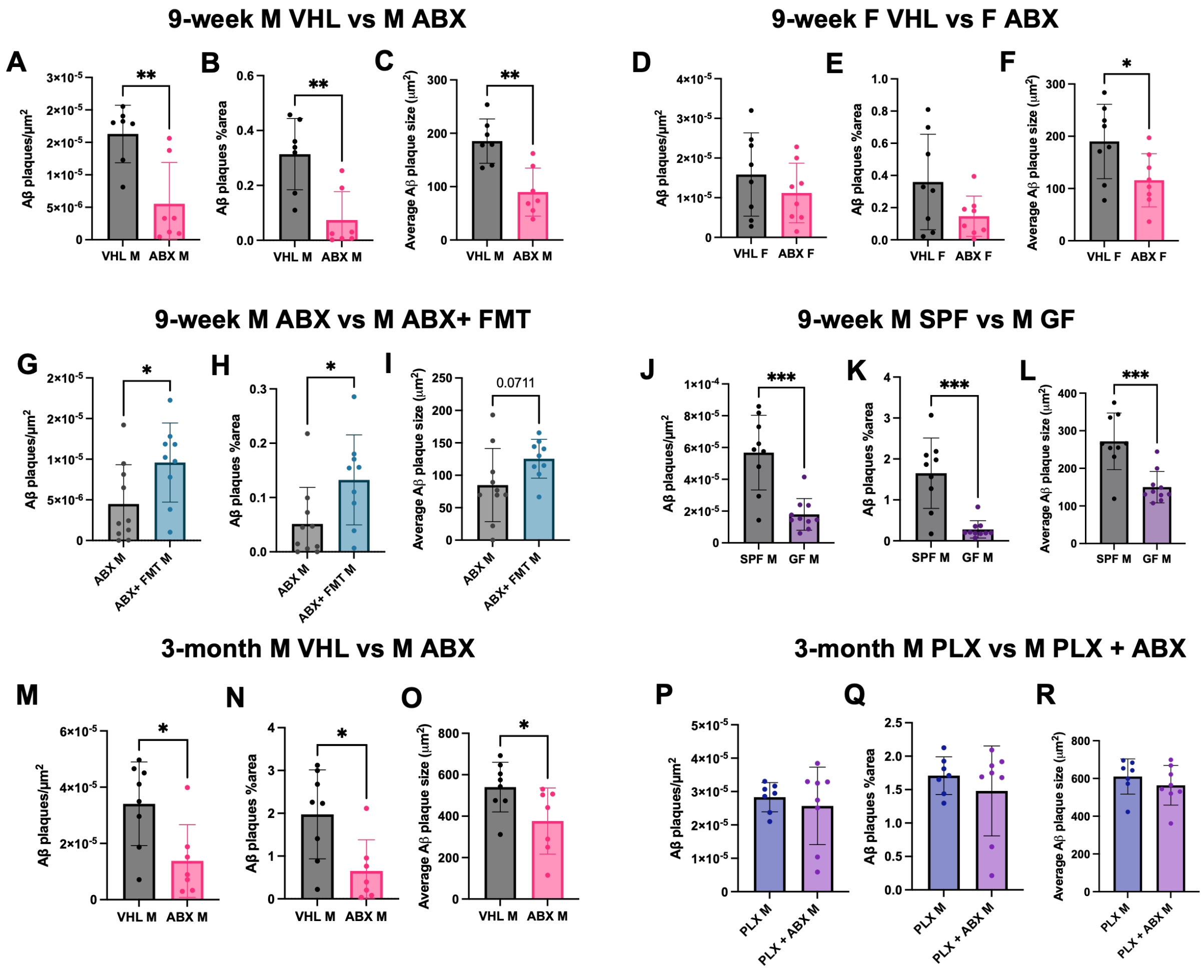
9-week M VHL vs M ABX
A
Aβ plaques/μm2
VHL M
ABX M
**
B
Aβ plaques %area
VHL M
ABX M
**
C
Average Aβ plaque size (μm2)
VHL M
ABX M
**
9-week F VHL vs F ABX
D
Aβ plaques/μm2
VHL F
ABX F
E
Aβ plaques %area
VHL F
ABX F
F
Average Aβ plaque size (μm2)
VHL F
ABX F
*
9-week M ABX vs M ABX+ FMT
G
Aβ plaques/μm2
ABX M
ABX+ FMT M
*
H
Aβ plaques %area
ABX M
ABX+ FMT M
*
I
Average Aβ plaque size (μm2)
ABX M
ABX+ FMT M
0.0711
9-week M SPF vs M GF
J
Aβ plaques/μm2
SPF M
GF M
***
K
Aβ plaques %area
SPF M
GF M
***
L
Average Aβ plaque size (μm2)
SPF M
GF M
***
3-month M VHL vs M ABX
M
Aβ plaques/μm2
VHL M
ABX M
*
N
Aβ plaques %area
VHL M
ABX M
*
O
Average Aβ plaque size (μm2)
VHL M
ABX M
*
3-month M PLX vs M PLX + ABX
P
Aβ plaques/μm2
PLX M
PLX + ABX M
Q
Aβ plaques %area
PLX M
PLX + ABX M
R
Average Aβ plaque size (μm2)
PLX M
PLX + ABX M
